# Transport‐Friendly Microstructure in SSC‐MEA: Unveiling the SSC Ionomer‐Based Membrane Electrode Assemblies for Enhanced Fuel Cell Performance

**DOI:** 10.1002/advs.202403647

**Published:** 2024-08-15

**Authors:** Min Li, Han Ding, Jingnan Song, Bonan Hao, Rui Zeng, Zhenyu Li, Xuefei Wu, Zachary Fink, Libo Zhou, Thomas P. Russel, Feng Liu, Yongming Zhang

**Affiliations:** ^1^ School of Chemistry and Chemical Engineering Frontiers Science Center for Transformative Molecules Center of Hydrogen Science Shanghai Key Lab of Electrical Insulation & Thermal Aging Shanghai Jiao Tong University Shanghai 200240 China; ^2^ School of Energy Power and Mechanical Engineering North China Electric Power University Beijing 102206 China; ^3^ Materials Sciences Division Lawrence Berkeley National Laboratory Berkeley CA 94720 USA

**Keywords:** fuel cell, humidity dependence, morphology‐transport‐performance, SSC/LSC‐MEA, transport‐friendly structure

## Abstract

The significant role of the cathodic binder in modulating mass transport within the catalyst layer (CL) of fuel cells is essential for optimizing cell performance. This investigation focuses on enhancing the membrane electrode assembly (MEA) through the utilization of a short‐side‐chain perfluoro‐sulfonic acid (SSC‐PFSA) ionomer as the cathode binder, referred to as SSC‐MEA. This study meticulously visualizes the distinctive interpenetrating networks of ionomers and catalysts, and explicitly clarifies the triple‐phase interface, unveiling the transport‐friendly microstructure and transport mechanisms inherent in SSC‐MEA. The SSC‐MEA exhibits advantageous microstructural features, including a better‐connected ionomer network and well‐organized hierarchical porous structure, culminating in superior mass transfer properties. Relative to the MEA bonded by long‐side‐chain perfluoro‐sulfonic acid (LSC‐PFSA) ionomer, noted as LSC‐MEA, SSC‐MEA exhibits a notable peak power density (1.23 W cm^−2^), efficient O_2_ transport, and remarkable proton conductivity (65% improvement) at 65 °C and 70% relativity humidity (RH). These findings establish crucial insights into the intricate morphology‐transport‐performance relationship in the CL, thereby providing strategic guidance for developing highly efficient MEA.

## Graphical abstract

This study highlights the crucial role of SSC‐PFSA ionomer as a cathodic binder in enhancing mass transport and performance in fuel cells, demonstrating superior structural and transport properties compared to LSC‐PFSA, thereby optimizing cell efficiency.

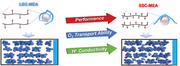

## 1 Introduction

The global development of hydrogen energy makes the proton exchange membrane fuel cells (PEMFCs) a crucial technology for delivering clean energy with high efficiency and high energy density.^[^ ^1^, ^2^ ^]^ PEMFCs have broad applications in transportation, distributed power stations, and commercial electronic devices.^[^ ^3^, ^4^ ^]^ PEMFCs are composed of cathode, anode, and proton exchange membrane (PEM). The cathode absorbs oxygen and catalyzes its reduction to H_2_O, constituting the most challenging electrochemical reaction in the PEMFCs.^[^ ^5^ ^]^ The cathode is regarded as the “engine” of the fuel cell, strongly impacting PEMFCs’ efficiency. Therefore, developing an advanced cathodic CL with enhanced oxygen diffusion, better water management, and higher catalytic efficiency is key.^[^ ^6^, ^7^ ^]^ Nevertheless, challenges persist in the CL, leading to elevated costs and diminished longevity for PEMFCs.^[^ ^8^, ^9^ ^]^

It's known that the mass transport losses can be a serious issue in the cathode CL, encompassing O_2_ transportation and proton conduction, which markedly limits the PEMFCs’ performance.^[^ ^10^, ^11^ ^]^ The mass transfer resistances induce substantial concentration polarizations, resulting in a decline in the cell's output voltage. Various effective approaches, such as triple‐phase boundary engineering, materials optimization, and porous electrode construction, have demonstrated promise in facilitating the microscopic multiphase mass transport processes in the CL.^[^ ^12^, ^13^ ^]^ Through the optimized electrode structure and configuration, the mass transfer resistances can be reduced, providing an optimal operational condition for electrochemical reactions in CL.^[^ ^14^ ^]^

Enhancing the proton conductivity is the first challenge, achievable by forming a better interconnected ionomer phase with an increased concentration of SO_3_^2−^ groups.^[^ ^15^, ^16^ ^]^ There are many polymers with different side chain structures, and we chose the more commonly used LSC‐PFSA and SSC‐PFSA structures for comparison.^[^ ^15^, ^16^, ^17^ ^]^ Compared with the LSC‐PFSA ionomer, the SSC‐PFSA ionomer proves to be a better cathodic CL binder, given its high ion exchange capacity (IEC) and therefore the exceptional proton conductivity.^[^ ^17^, ^18^ ^]^ Another challenge lies in O_2_ transport, imposing limitations on both the efficiency and longevity of PEMFCs.^[^ ^19^ ^]^ O_2_‐transport resistances originate from multiple processes, which include gas diffusion in large pores (mainly molecular diffusion, R_MD_), gas diffusion in small pores (mainly Knudsen diffusion, R_Kn_), and O_2_ dissolution and diffusion in ionomer (R_CL,ion_).^[^ ^20^ ^]^ Tremendous efforts have been devoted to mitigating the O_2_ transport hindrances in CL, involving material optimization (optimizing catalysts and ionomers combinations) and morphology optimization, ultimately facilitating the O_2_ diffusion and diminishing overpotential during the PEMFCs’ working condition.^[^ ^21^, ^22^ ^]^

Simultaneously optimizations of proton conductivity and O_2_ transport in the cathode is the ultimate goals, a critical yet challenging aspect of fuel cell technology. We specifically aim to find an optimal balance between a more well‐interconnected ionomer phase and the desired O_2_ diffusion at multiple‐length‐scale pores. This balance is vital for advancing the performance and efficiency of PEMFCs. Therefore, selecting a high proton‐conductivity ionomer with outstanding pore‐forming ability and decreased Pt/ionomer interfacial resistance is of great importance.^[^ ^22^ ^]^ There has been a growing emphasis on finding the optimal cathodic binders.^[^ ^23^, ^24^ ^]^ Binding a desired ionomer with the catalysts not only reduces the O_2_ transport hindrances but accelerates the deliveries of protons in the CL, thereby lowering the overpotential during PEMFCs’ working condition.^[^ ^16^ ^]^ With the usage of SSC‐PFSA ionomer in recent years, optimizing the structural and performance characteristics of PEMFCs has become more feasible.^[^ ^25^, ^26^ ^]^

The LSC‐PFSA and SSC‐PFSA ionomers are the major materials employed as cathodic CL binders, delivering satisfactory performance and stability. However, they behave differently based on different cell operating conditions. Hence, our study focuses on the distinctive behaviors of LSC‐PFSA and SSC‐PFSA ionomers, the predominant cathodic CL binders, under various operating conditions. A quantified and well‐established morphology‐transport‐performance relationship is in urgent need, as the effect of CL morphology on proton transport and O_2_ diffusion is hard to be adequately quantified. Our research aims to fill this critical gap, thereby contributing significantly to the field of fuel cell technology.

In this study, the influences of morphology on proton transport and O_2_ diffusion have been quantitatively analyzed. We visualized the complex multi‐length‐scale morphology of the cathodic CL using LSC‐PFSA and SSC‐PFSA ionomers and conducted the PEMFCs’ performance test by evaluating the voltage losses and mass transport resistances under various humidity conditions. The multi‐step mass transport processes within the CL are effectively decoupled and quantified to the large pore diffusion, small pore diffusion, and ionomer diffusion. We demonstrate that SSC‐PFSA binder affords better transport properties, proton conductivities, and superior power density, and can execute improved water management in MEA, especially at 65 °C and 70% RH. A clearly established CL morphology picture is obtained, and a more efficient triple interface can be reached in SSC‐MEA. These results underscore the critical influence of ionomer binders on PEMFC performance, shedding light on the complex interplay between morphology‐transport‐performance. Our study provides pivotal insights into optimizing CL design for enhanced fuel cell functionality.

## 2 Results and Discussion

**Figure 1:**
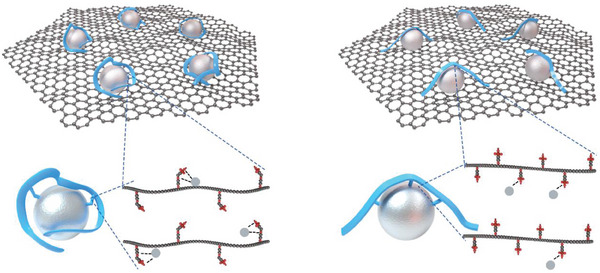
The schematic illustration of the cathode for the LSC‐MEA (left) and SSC‐MEA (right). Structural representation of the LSC‐PFSA (left) and SSC‐PFSA (right), respectively. Blue, ionomer fibrils; Silvery, Pt particles; Red, O; brown, S; gray, CF_x_.

We fabricated the two MEAs (noted as LSC‐MEA and SSC‐MEA) using the perfluoro‐2‐(2‐sulfonic acid ethoxy) propoxy endcapped LSC‐PFSA and SSC‐PFSA ionomers^[^ ^27^ ^]^ as the binders in the CL, and a schematic illustration is shown in **Figure** **1**. Although the SSC‐PFSA ionomer, having a higher SO_3_^2−^ group concentration, results in more binding site to Pt catalyst particles, the short‐side‐chain in the SSC‐PFSA ionomer is more rigid due to the lack of an ether group interacting with Pt, as evidenced in subsequent morphology characterization and molecular dynamics simulation. The side chain and backbone need to cohesively adjust the conformation to form the ionomer‐Pt binding. Thus, the SSC‐PFSA adsorption to Pt nanoparticles can be weaker compared to LSC‐PFSA.^[^ ^28^, ^29^ ^]^ The prepared routing and corresponding pictures of the MEA is diagrammed in Figures S1 and S2 (Supporting Information).

**Figure 2:**
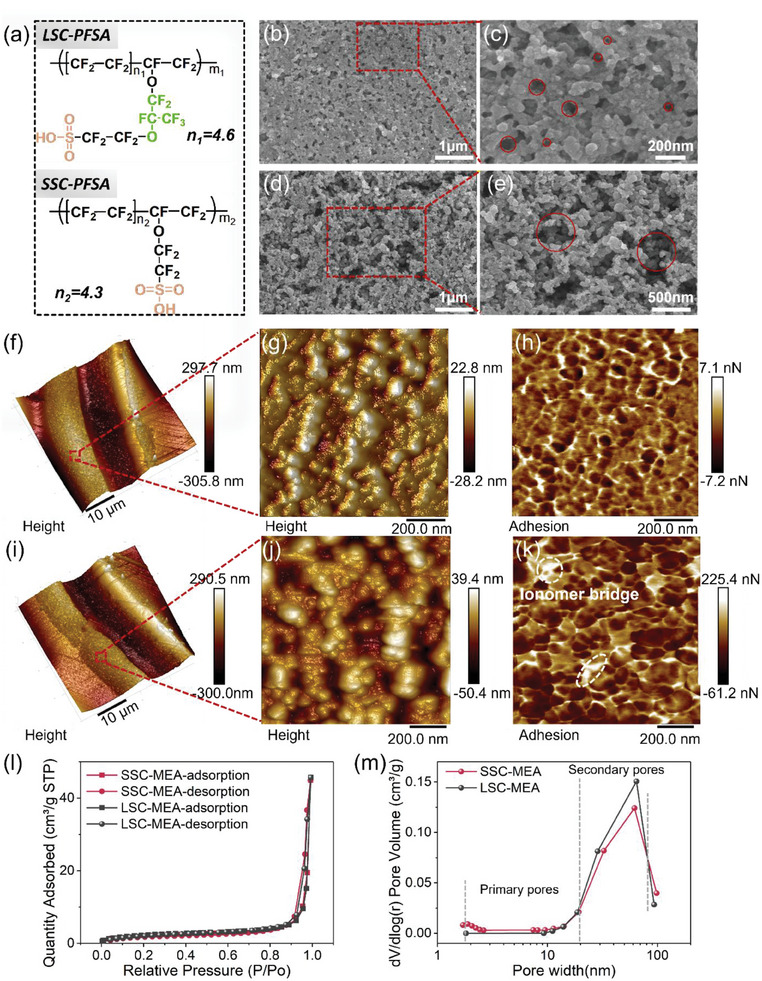
a) Structural formulas of the LSC‐PFSA and SSC‐PFSA. SEM images of the cathodic surface of b,c) LSC‐MEA and d,e) SSC‐MEA. f) the cross‐section AFM morphography of LSC‐MEA. g) The amplifying height image and h) the adhesion image of the cathode of LSC‐MEA. i) The whole cross‐section AFM morphography of SSC‐MEA. j) The amplifying height image and k) the adhesion image of the cathode of SSC‐MEA. l) N_2_ adsorption and desorption isotherms and m) pore‐width distribution of the LSC‐MEA and SSC‐MEA.

The chemical structure of the LSC‐PFSA and SSC‐PFSA is depicted in **Figure** **2**a. First, we conducted a comprehensive analysis of slurry rheology, focusing on its impact on ink properties and the coating process, as illustrated in Figure S3 (Supporting Information).^[^ ^30^ ^]^ Both slurries displayed non‐Newtonian, shear‐thinning characteristics (shear‐thinning index n < 1), with shear viscosity (η) declining and shear stress (σ) escalating alongside shear rate. LSC‐MEA's lower shear‐thinning index suggested more pronounced aggregation disruption under shear, reducing viscosity. Conversely, SSC‐MEA's higher index indicated greater structural resilience to shear forces. Our three‐interval thixotropy test (3ITT) mimicked the coating sequence: low shear (1 s^−1^) forming a weak gel network, high shear (100 s^−1^) disrupting this network and reducing viscosity, and a recovery phase showing viscosity returning to baseline (1 s^−1^) (Figure S3b, Supporting Information). SSC‐MEA demonstrated superior structural recovery post‐shear, indicating a more robust network compared to LSC‐MEA. In Figure S3c,d (Supporting Information), both slurries showed higher storage modulus (G′) than loss modulus (G″), with a dominant solid‐like character evidenced by G′ exceeding G″ in the linear viscoelastic region. Under high oscillation strain, viscous behavior prevailed. SSC‐MEA's higher G′ and G″ values suggested increased viscosity and elasticity, and its yield stress (σ_*y*_) was notably higher, crucial for particle stability in suspension. Frequency sweeps revealed SSC‐MEA's elasticity was less affected by frequency changes, unlike LSC‐MEA, which showed relatively stable elasticity and viscosity in response to frequency variations (Figure S3e, Supporting Information).

The cathodic surface morphology is probed by scanning electron microscopy (SEM), while the cross‐section morphology is investigated by atomic force microscopy (AFM). Figure S4 (Supporting Information) reveals that LSC‐MEA is characterized by longer and less frequent cracking, in contrast to SSC‐MEA, which exhibits a greater number of smaller cracks. This discrepancy in crack formation could be attributed to the differences in the Pt/C aggregate structures within the respective slurries. LSC‐MEA exhibits a porous surface with ≈ 50–100 nm voids. These voids are sparsely distributed, thus limiting their connectivity (Figure 2b,c; Figure S5a–d, Supporting Information). In contrast, SSC‐MEA displays a “coral” texture with multi‐length‐scale porous morphology (<1 µm) on the cathodic surface (Figure 2d,e; Figure S5e–h, Supporting Information). The porosity of SSC‐MEA looks much higher than that in LSC‐MEA. Despite using the same ionomer‐to‐carbon (I/C) ratio in MEA preparation, the LSC‐PFSA ionomer seemingly sticks to Pt/C catalyst tightly. Oppositely, the SSC‐PFSA coalesces with the catalytic phase to form a more porous structure. This feature formation can be attributed to higher rigidity of backbones^[^ ^28^ ^]^ and greater contraction of SSC‐PFSA ionomer during the solvent evaporation process^[^ ^31^ ^]^ compared with LSC‐PFSA. The more flexible side chain in the LSC‐PFSA ionomer leads to higher conformation flexibility. During the CL condensation, the ionomer phases merge to form a compact CL.

The overall cross‐section topography with a size of 40 µm × 40 µm is captured, uncovering a well‐defined trilaminar structure of the LSC‐MEA and SSC‐MEA, consisting of the anode, membrane (comprising bilateral ionomer layers and central reinforcement) and cathode (Figure 2f,i; Figure S6, Supporting Information). In the detailed local view of cathodic cross‐section topography, 3 µm × 3 µm, and 1 µm × 1 µm maps were captured. In height images, SSC‐MEA shows larger height difference due to the more abundant large voids compared to LSC‐MEA (Figure 2g,j; Figure S7a,c, Supporting Information). Viscoelastic mode AFM is used to distinguish PFSA ionomer since it is a soft material and has a higher adhesion with the AFM tip. Obvious phase separation in the cathode can be seen. The Pt/C catalyst phase appears dark in adhesion maps because of their low values of adhesive forces.^[^ ^32^ ^]^ The bright area in adhesion map tracks the distribution of PFSA ionomer.^[^ ^33^ ^]^ In LSC‐MEA, ionomers isolate the Pt/C aggregates and form interconnected fibril morphology with a diameter of ≈20 nm (Figure 2h; Figures S7b and S8, Supporting Information). In SSC‐MEA, the interconnected fibril morphology is also seen. However, the thinner bright fibrils, with a slender diameter of ≈10–15 nm (Figure S9, Supporting Information), are observed to form a better‐interconnected ionomer network with the same I/C ratio in the two CLs (Figure 2k; Figure S7d, Supporting Information). The slender fibrils can dramatically reduce the resistances from O_2_ diffusion in the ionomer fibrils. Besides, we see slightly less bright regions acting as “ionomer bridges” that link the fibrils to form a more continuous proton‐conductive network. These results imply that the a more efficient proton transport in SSC‐MEA is expected in morphology basis.

To further explore the pore structure, the N_2_ adsorption and desorption isotherms were conducted (Figure 2l). The Brunauer‐Emmett‐Teller (BET) surface area of MEA is normalized by its weight to afford comparable data. The surface area for SSC‐MEA is 9.4 m^2^ g_MEA_^−1^, larger than that of LSC‐MEA (7.9 m^2^ g_MEA_^−1^). The cumulative volume of pores (>1.7 nm width) in SSC‐MEA is 0.079 cm^3^ g_MEA_^−1^, also larger than that in LSC‐MEA (0.069 cm^3^ g_MEA_^−1^). The pore‐width distribution profile reveals the size‐dependent pore volume information (Figure 2m). The primary pore volume (2–20 nm) of SSC‐MEA exceeds that of LSC‐MEA. The secondary pore volume (20–80 nm) of SSC‐MEA is smaller than that of LSC‐MEA. Even so, in the larger size region (>80 nm), SSC‐MEA displays a higher pore volume. It is important to note that N_2_ adsorption‐desorption test cannot probe the pore structures larger than 100 nm, and in the larger size region, SSC‐MEA displays higher porosity as seen in the SEM results. These results reveal the interaction feature between the bonded ionomers and the Pt/C catalysts. LSC‐PFSA ionomer is more readily to fill the primary pores, which is associated with its more flexible conformation and more hydrophobic properties in comparison to SSC‐PFSA.^[^ ^34^, ^35^ ^]^ The hydrophilic side chain would attach to Pt nanoparticles, and the hydrophobic backbone would naturally self‐assemble and interact with hydrophobic carbon spheres, leading structure reconstruction that fills the nanosized pores. This feature would significantly hinder the O_2_ transport in adjacent to Pt particles since it is agglomerated by LSC polymer healing. On the contrary, SSC‐PFSA ionomers fill fewer primary pores, indicating more hydrophobic primary pores and thus better water‐draining management, conducive to the O_2_ diffusion to the Pt surface. In consideration of the BET and SEM results, although LSC‐MEA shows more 20–80 nm mesopores, the SSC‐MEA displays better pore hierarchy in <1 µm size region, providing large flux and lower resistance when O_2_ permeates from GDL to Pt sites and leading to better O_2_ transport in SSC‐MEA.

**Figure 3:**
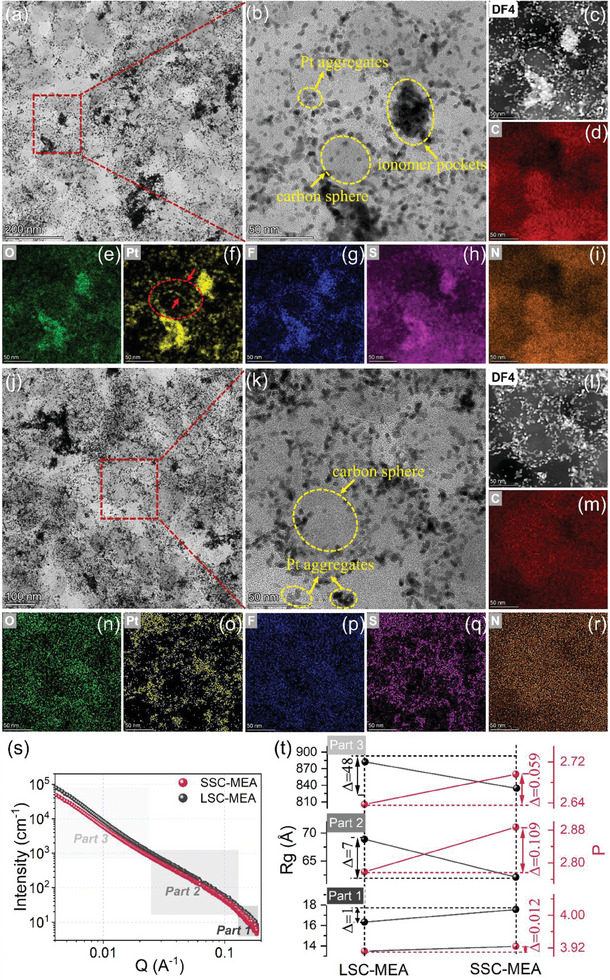
Morphology and structural characterizations. a,b) TEM images, c) HAADF‐STEM images, and d–i) corresponding element mappings of the cathode of LSC‐MEA; j,k) TEM images, l) HAADF‐STEM images, and m–r) corresponding element mappings of the cathode of SSC‐MEA; s) 1D GISAXS profiles and () the values of the *R_g_* and Porod slope of the LSC‐MEA and SSC‐MEA.

We then visualized the phase separation interface of ionomer/Pt/carbon using transmission electron microscopy (TEM). Obvious carbon agglomerates and Pt nanoparticle clusters are observed in both LSC‐MEA and SSC‐MEA (**Figure** **3**a,j; Figure S10, Supporting Information). The samples were beforehand placed in the humidor for 48 h. The Pt nanoparticles are more uniformly distributed in the SSC‐MEA globally, which may be attributed to the weaker interaction between the SSC‐PFSA ionomer and Pt. However, in LSC‐MEA, more Pt nanoparticle aggregates are seen, indicating that the LSC‐PFSA ionomer could peel off Pt nanoparticles from the carbon support to form Pt aggregates (Figure S10, Supporting Information). The ionomer attaches with Pt via the O‐Pt interaction, and thus the LSC‐Pt interaction is thus stronger.^[^ ^28^ ^]^ The isolated Pt clusters in ionomer pockets without carbon support could not take charge carriers out, leading to a “dead area” in the electrochemical reaction. The amplifying TEM images and corresponding high‐angle angular dark field‐scanning transmission electron microscopy (HAADF‐STEM) characterizations were conducted to provide better phase contrasted images (Figure 3b,c,k,l). It is seen that the carbon spheres are covered by ionomers mixed with Pt particles. Additional element mappings reveal the C, N, O, Pt, F, and S distributions (Figure 3d–i,m–r). The similar distribution of the Pt, O, and S elements in the mappings verifies the interaction between Pt and side chains. The phase‐separated morphology is further analyzed by grazing incidence small‐angle X‐ray scattering (GISAXS) using synchrotron scattering beamline facilities (Figure S11, Supporting Information). The 2D GISAXS patterns were converted to 1D profiles, where the three key structures are identified (Figure 3s). The 1D profiles are fitted in three levels (part 1, part 2, part 3) using the Guinier‐Porod method. The fitted values of Porod slopes (*P*) in LSC‐MEA are 3.91 for part 1, 2.78 for part 2, and 2.64 for part 3, respectively. The *P* values for SSC‐MEA show a similar trend but slightly larger values (Figure 3t), which suggest a slightly more condensed aggregates of Pt clusters (Part 2) and carbon aggregates (Part 3).^[^ ^36^ ^]^ The radius of gyration (*R_g_*) for LSC‐MEA is fitted to be 16 Å for part 1, 69 Å for part 2, and 882 Å for part 3, respectively. In SSC‐MEA, these values are 17 Å for part 1, 62 Å for part 2, and 834 Å for part 3 respectively. The Part 1 and Part 2 scattering originate from Pt single nanoparticle and Pt clusters. Thus, a reduced aggregation and better distribution of Pt nanoparticles are obtained in SSC‐MEA. The high scattering intensity observed in part 3 was attributed to the carbon aggregates interfaces, and the *R_g_* of SSC‐MEA for Part 3 is smaller, indicating a smaller average size of carbon agglomerates. The reduced size and more condensed packing of carbon particles should result from the more effective binder assembling during CL formation, leading to better charge transport channels and higher pore volumes.

**Figure 4:**
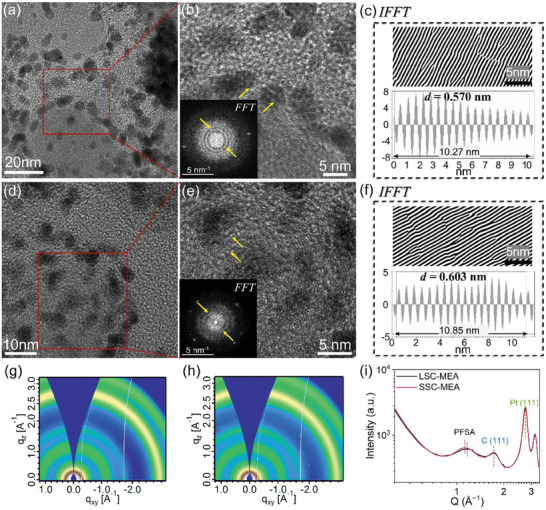
HRTEM images and their corresponding *IFFT* results of the cathodic cross‐section of a–c) LSC‐MEA and d–f) SSC‐MEA, the inset of Figure (b,e) is the corresponding *FFT* patterns; 2D GIWAXS images of the g) LSC‐MEA and h) SSC‐MEA and i) their corresponding 1D curves.

The close interaction between Pt nanoparticles, carbon substrates, and ionomers was studied by high‐resolution transmission electron microscopy (HRTEM) in cross‐section geometry. It is seen that the ionomers are in close contact with Pt particle and carbon support (**Figure** **4**a,b,d,e). Both LSC‐PFSA and SSC‐PFSA ionomers form ionomer assemblies, covering the Pt nanoparticles and carbon spheres. There is a visible tiny space (<1 nm) between the ionomer strands on the Pt particle surface, which is a clear visualization of the triple‐phase interface. The SSC‐PFSA in MEA shows more straight‐line patterns, which is due to a more rigid chain conformation. Diffraction patterns are generated by fast Fourier transformation (*FFT*) (inset of Figure 4b,e). The inverse *FFT* (*IFFT*) operation was carried out to generate the lattice fringes, where the line profiles were made to calculate the lattice spacings (d) (Figure 4c,f). The exact interplanar spacings are ≈0.570 nm for LSC‐PFSA and ≈0.603 nm for SSC‐PFSA. The slightly large distance may be attributed to enlarged ionic domain spacing^[^ ^31^ ^]^ and surface adsorption of ionomers, in which the SSC‐PFSA interchain interaction is counter‐balanced by the surface adsorption force to enlarge the d spacing. The lattice spacings are further verified using the grazing incidence wide‐angle X‐ray scattering (GIWAXS) method (Figure 4g–h). The 2D GIWAXS profiles were converted to 1D curves by a circular average the intensities. The peaks at 2.75 and 3.17 Å^−1^ correspond to the interplanar spacing of Pt {111} and {200}. The peak at 1.73 Å^−1^ represents the graphite {002}. These diffractions coincident in the two samples due to the same Pt/C catalysts. The peaks at 1.16 Å^−1^ (for LSC‐MEA) and 1.13 Å^−1^ (for SSC‐MEA) represent the space between the backbones of LSC‐PFSA and SSC‐PFSA ionomers, respectively, confirming the slightly closer stackings of LSC ionomer chains. The interaction between Pt/C catalyst and ionomer is the last step that influences O_2_ diffusion. The formation of the triple‐phase point is critical to support the O_2_ reduction reaction by feeding in sufficient O_2_ and proton to the Pt active sites. It is difficult to quantify the quality and volume ratio of triple‐phase points in LSC‐MEA and SSC‐MEA. Nevertheless, we know that perfluoro‐sulfonic acid ionomers have better O_2_ permeability as the binders compared to other ionomer materials,^[^ ^37^ ^]^ and in the SSC‐MEA case, the slightly larger interchain spacing could further enhance O_2_ diffusion in the crystalline regions since the oxygen molecule has a smaller size (≈0.296 nm).

**Figure 5:**
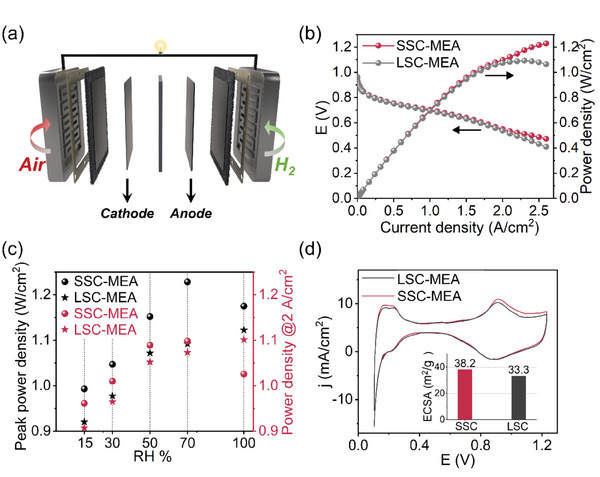
H_2_‐Air fuel cell performance. a) Scheme of the single cell. b) Polarization curves of the LSC‐MEA and SSC‐MEA at 65 °C and RH 70%. c) Summaries of the peak power density and power density at 2 A cm^−2^. d) CV curves and the corresponding ECSA values of the LSC‐MEA and SSC‐MEA at 65 °C and 70% RH.

To establish the morphology‐performance relationship, H_2_‐Air single‐stacked fuel cell devices with an area of 25 cm^2^ were fabricated and tested at 65 °C and 150 kPa back pressure (**Figure** **5**a). The polarization curves at 100%, 70%, 50%, 30%, and 15% RH were collected (Figure 5b; Figure S12, Supporting Information). The I/C ratio is set at 0.7 with Pt loading of 0.35 mg cm^−2^ in the cathode and 0.05 cm^2^ in the anode, which is in accordance with the samples used in structure characterization. The results reveal that SSC‐MEA exhibits a higher peak power density over all the humidity conditions, and at 70% RH, it reaches 1.23 W cm^−2^, which is higher than that of LSC‐MEA (Figure 5c). The highest power density at 2 A cm^−2^ (1.10 W cm^−2^) is also existed in the SSC‐MEA at RH 70%. The cyclic voltammetry (CV) is employed to assess the electrochemical active surface area (ECSA) at 70% RH. A higher ECSA value is seen for SSC‐MEA (Figure 5d), suggesting the weaker shielding effect of SSC‐PFSA ionomer on Pt nanoparticles, and contributing to improved cell performance. Moreover, the hydrogen penetration current density and internal resistance of the SSC‐MEA are smaller than those of LSC‐MEA, favorable for the cell performance (Figure S13, Supporting Information).

**Figure 6:**
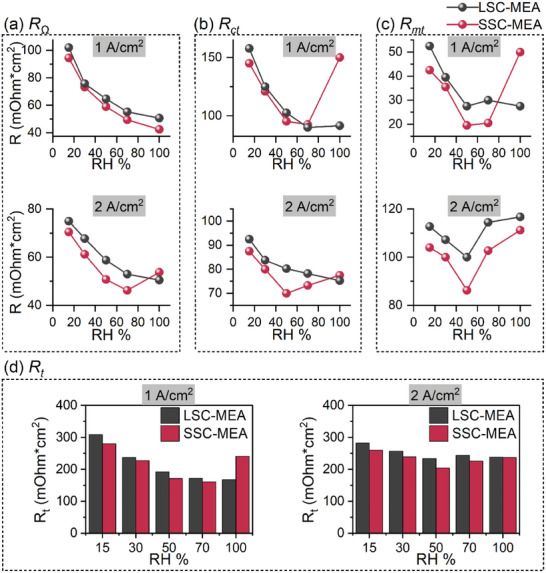
Kinetics and transport properties. a) The fitting parameters of Nyquist plots: a) R_Ω_, b) R_ct_, c) R_mt_ at 1, and 2 A cm^−2^. d) The summaries of the R_t_.

Electrochemical impedance spectroscopy (EIS) was conducted during the working conditions of various humidities (15%, 30%, 50%, 70%, and 100% RH) and at certain current densities (1 A cm^−2^, 2 A cm^−2^) to analyze the real resistances in transport and electrode kinetic processes (Figures S14 and S15, Supporting Information). The EIS curves were fitted to an equivalent circuit model that contains Ohmic resistance (R_Ω_, the intersection observed at the high‐frequency response arcs of the spectra along the x‐axis related to inherent resistance of cell), charge‐transfer resistance (R_ct_, primarily dictated by the electrode kinetic loss of the cathode at medium‐frequency response arcs of the spectra), mass‐transport resistance (R_mt_, attributed to the O_2_ diffusion to the reaction sites and observed at low‐frequency response arcs) and constant phase elements (CPE1 and CPE2).^[^ ^38^, ^39^ ^]^ The as‐fitted results are summarized in **Figure** **6**. The R_Ω_ values of SSC‐MEA are lower than those of LSC‐MEA at various humidities, suggesting enhanced electroconductivity (Figure 6a). At 1 A cm^−2^ current density and low to moderate humidity, the R_ct_ values of SSC‐MEA are slightly lower than LSC‐MEA at most situations, indicating the slightly lower kinetic losses in SSC‐MEA (Figure 6b). However, at full humidity condition, the SSC‐MEA exhibits a dramatic increase of R_ct_, forming a distinct contrast to the slight increase of LSC‐MEA. This result aligns with performance behaviors. We speculate that this phenomenon is related to the fast dilution of the local proton and O_2_ concentrations in water‐swelling SSC‐PFSA during the intense electrochemical reactions. Because the SSC‐PFSA with a high IEC often appears much more water‐uptake ability than the LSC‐PFSA.^[^ ^40^ ^]^ As the reaction progresses, both ionomers absorb a considerable amount of water, reaching equilibrium. Hence at 2 A cm^−2^, the R_ct_ of SSC‐MEA is slightly higher than that of LSC‐MEA, attributed to the stable and slightly lower proton and O_2_ concentrations. The R_mt_ values of SSC‐MEA are lower than those of LSC‐MEA, revealing more efficient O_2_ diffusion from GDL to Pt active sites in SSC‐MEA at operating conditions (Figure 6c). It is worth noting that the decrease in mass transport resistance (R_mt_) for SSC‐MEA aligns with the observed superior hierarchical‐porous structure and thinner ionomer fibrils. Nevertheless, the R_mt_ at 1 A cm^−2^ of SSC‐MEA is hugely increased when the humidity improves to 100%. The abundant water produced by initially violent electrochemical reaction was chiefly taken up by the PFSA ionomer at medium current region, leading the excessive swelling in SSC‐PFSA, and thus increasing the length of the O_2_ diffusion. As the reaction goes on and structural reforming finishes, the water produced by violent electrochemical reaction is drained away through the pore structure. Thus, the mass transport of SSC‐MEA at 2 A cm^−2^ exhibits obvious advantage. The values of total resistances (R_t_) are summarized in Figure 6d. The R_t_ values of SSC‐MEA are lower than those of LSC‐MEA in most conditions, especially at low to moderate humidities (Figure 6d). The EIS results clarify the specific contributions of the R_Ω_, R_ct,_ and R_mt_ to the voltage losses during the cell operating process, and confirm the effects from pore structure or ionomers on the cell performance. The SSC‐MEA shows lower resistances, which is exceedingly in favor of the cell performance. A comparative analysis of the performance with existing literature is shown in Table S1 (Supporting Information). The SSC‐MEA as‐prepared in this research shows some advantages in terms of power density and total resistances.

**Figure 7:**
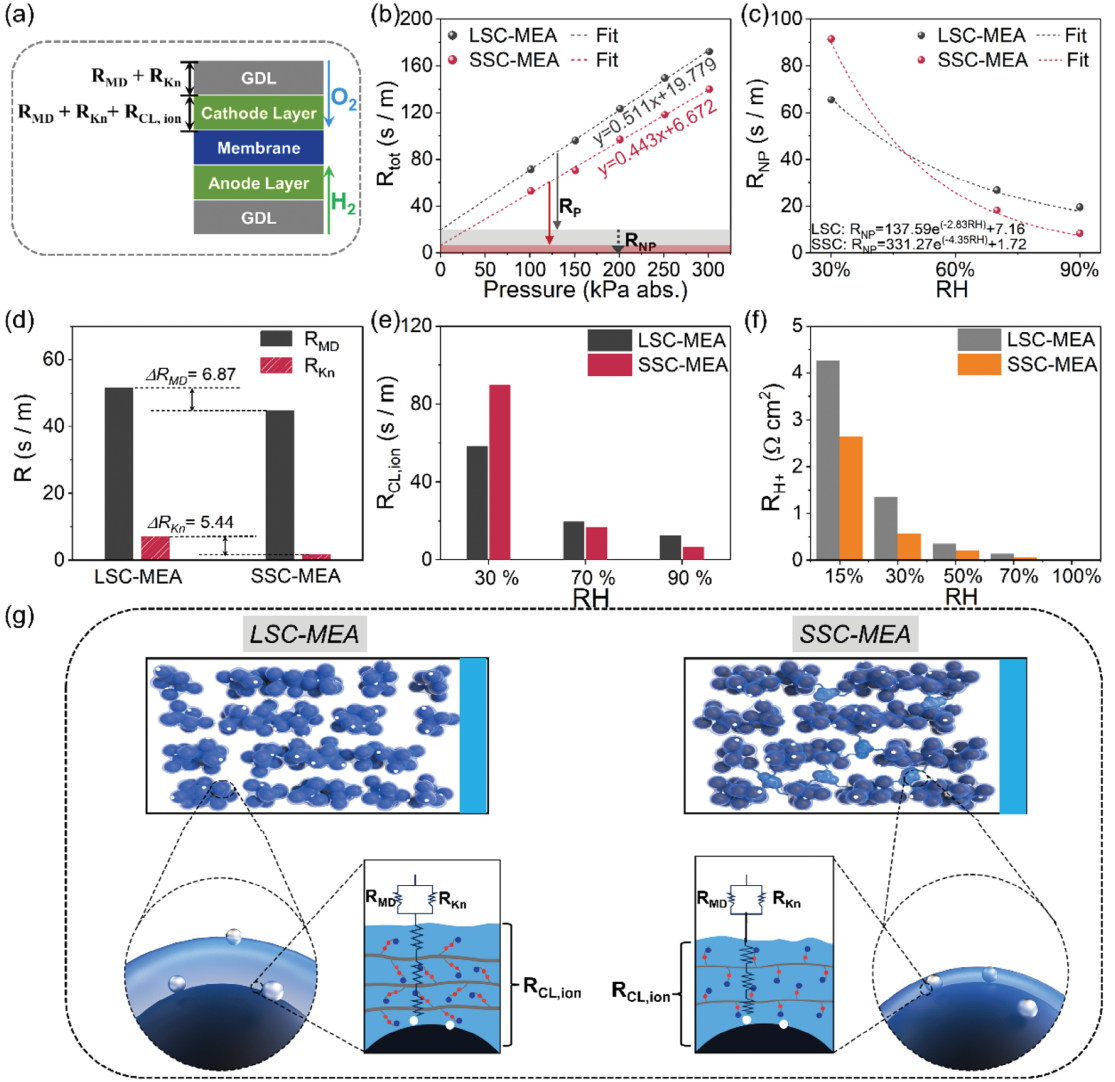
Mass transport. a) The diagram of local O_2_ transport resistance in the MEA. b) Total resistance at 90% RH and different back pressure. c) R_NP_ resistances at different humidity. d) Values of as‐fitted molecular diffusion and Knudsen diffusion resistances at 101 kPa abs. and e) ionomer diffusion resistance at 101 kPa abs. and various humidities. f) Proton conductivity comparison at different humidities. g) Scheme of the oxygen transport in the cathode of the LSC‐MEA (left) and SSC‐MEA (right) at 70% RH.

To profoundly understand the transport mechanisms and transport‐performance coupling, in‐depth analysis of O_2−_ transport and proton‐conduction processes are pivotal. O_2_ transport is linked to the diffusion in porous media and permeation through the ionomer cladding on Pt nanoparticles. Thus, a more detailed analysis is imperative. **Figure** **7**a and Table S2 (Supporting Information) illustrate the mechanism of O_2_ transport, including gas‐phase diffusion in large pores (R_MD_), subsequent gas‐phase diffusion in small pores (R_Kn_), and ultimate dissolution and diffusion in ionomers (R_CL,ion_). The methods to separate the O_2_ transport resistances is shown in Table S3 (Supporting Information). We conducted the local O_2_ transport experiments using the 1% O_2_ in N_2_ gas and pure H_2_ to assess the O_2_‐limited fuel cell performance, thereby evaluating the transport resistance of O_2_ in limiting the device performances.^[^ ^41^ ^]^ In this experiment, the structural reforming resulting from the water produced during the reaction is excluded, given the minimal occurrence of electrochemical reactions. Unlike the mass transport resistance in the EIS, the local O_2_ transport resistance is directly relative to the intrinsic structure of the CL and polymer's inherent attribute at various humidities. A semi‐empirical model has been developed to analyze the total O_2_ transport resistance (R_tot_) in MEA, which decouples the total resistance into R_MD_, R_Kn_, and R_CL, ion_.^[^ ^20^ ^]^ Various back pressure and humidity conditions were employed in PEMFACs’ performance test to obtain the detailed values shown in Figure 7b,c. The results of limiting current densities (J_L_) and as‐fitted resistances are shown in Tables S4 and S5 (Supporting Information). Adjusting the back pressure is in order to separate the pressure‐dependent resistance R_P_ (related to the slope, primarily dominated by R_MD_) from the R_tot_ (Figure 7b). The R_tot_ values are extremely lower in SSC‐MEA at various pressures and 90% RH. Meanwhile, the R_MD_ value of SSC‐MEA at 90% RH and 101 kPa (absolute pressure) is 44.74 s m^−1^, appears lower than that of LSC‐MEA (51.61 s m^−1^), which is due to more large pores in SSC‐MEA as evidenced by SEM and BET results (Figure 7d). The results suggest superior O_2_ diffusion in SSC‐MEA at 90% RH.

It's known that the gas‐phase diffusion is hardly affected by humidity changing.^[^ ^20^ ^]^ We then adjust the humidities to separate the humidity‐dependent resistance (dominated by R_CL,ion_) from nonpressure‐dependent resistance R_NP_ (Figure 7c). In a low‐humidity region (30% RH), R_NP_ exhibits a smaller value in LSC‐MEA. Conversely, in a high‐humidity region (70% and 90% RH), R_NP_ exhibits a smaller value in SSC‐MEA. In order to explore the underlying reasons, further separation is needed. The fitted values of R_Kn_ and R_CL,ion_ are summarized in Figure 7d,e. The R_Kn_ value for SSC‐MEA is 1.72 s m^−1^, representing a significant reduction than that of LSC‐MEA (7.16 s m^−1^). This is a key value that confirms the desired hierarchical pore structure in SSC‐MEA provide superior O_2_ transport channels to reach to the reaction site frontiers. The R_CL,ion_ values progressively reduce with increasing humidity (Figure 7e). The R_CL,ion_ value of SSC‐MEA increases 1.53‐fold compared to that of LSC‐MEA at 30% RH, attributed to the greater contraction of the ionic domain spacing and intercrystallite domain spacing under lower humidities compared to the LSC‐PFSA, hindering the transport of the O_2_.^[^ ^21^, ^31^, ^42^ ^]^ This has been verified in our group's previous work.^[^ ^29^ ^]^ However, under the practical operating conditions of the PEMFCs, the plentiful water generated through electrochemical reactions serves to mitigate such contractions, so that minimal losses resulting from concentration polarization were observed in the polarization curve at lower humidities. At higher humidities, R_CL,ion_ values of SSC‐MEA decrease 0.84‐fold and 0.53‐fold compared to those of LSC‐MEA, respectively. The O_2_ diffusion of SSC‐MEA appears much more sensitive to humidity. There are two reasons: 1) the larger expansions of domain spacing and intercrystallite domain spacing in the SSC‐PFSA fiber at higher humidity and more compact shrinkages at lower humidity due to increased sensitivity of SSC‐PFSA to humidity; 2) Considering the increased water‐uptake ability of SSC‐PFSA ionomer under higher humidity, it is reasonable to conclude improved O_2_ solubility and diffusion in the water or moderately swollen ionomers, rather than hydropenic ionomers.^[^ ^43^ ^]^ The proton conductivity in the CL was assessed using the H_2_‐N_2_ impedance spectroscopy (Figure 7f; Figure S16, Supporting Information).^[^ ^44^ ^]^ SSC‐MEA exhibits superior proton‐conductive capabilities compared to LSC‐MEA at various humidities, owing to the higher concentration of SO_3_^2−^ groups and a better‐developed ionomer network. With increasing humidity, proton‐conductive resistance (R_H+_) experiences a significant reduction, and the difference in R_H+_ between the two MEAs gradually diminishes. Especially under full humidity conditions, the proton conduction advantage from SSC‐PFSA ionomer with higher IEC is attenuated and the R_H+_ value simultaneously approaches zero, suggesting the active involvement of water in proton conduction. The pathways of O_2_ transport and proton conduction are illustrated in Figure 7g. Based on above results coupled with morphology characteristics, three significant findings emerge: 1) a better triple phase point is formed in SSC‐MEA that more efficient O_2_ transport to reaction sites takes place; 2) the desired hierarchical pore structure and thinner ionomer fibrils provide less‐obstructed pathways for oxygen transport; 3) wetting of the CL could significantly enhance the O_2_ transport to reaction sites, possibly attributed to improved O_2_ solubility in water or water‐swelling ionomers.

**Figure 8:**
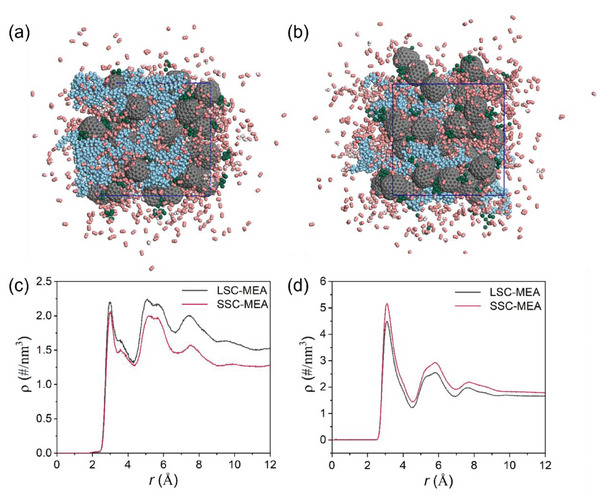
The molecular models of the CL bonded with a) LSC‐PFSA and b) SSC‐PFSA at 70% RH and 250 kPa abs. Pressure. Blue, PFSA; green, Pt; grey, C; pink, O; white, H. c) The atom density profiles of Pt‐SC. d) The atom density profiles of Pt‐O_2_.

Based on the intricate correlation of morphology‐transport performance, we subsequently carried out molecular dynamics (MD) simulations to elucidate the effects of the side‐chain structure on the catalyst layer. The chemical model of LSC‐MEA and SSC‐MEA, consisting of Pt/C catalysts and ionomer assemblies, were built in a simplified manner with two different views at 65 °C, 70% RH, and 250 kPa abs. pressure, consistent with optimum I/C ratios and operating conditions (**Figure** **8**a,b; Figures S17 and S18, Supporting Information). The microstructure snapshots reveal that the LSC‐PFSA are more readily to self‐aggregate than SSC‐PFSA, leading to the formation of thicker assemblies as seen in the AFM. Conversely, SSC‐PFSA slightly extended into the voids, thereby establishing a well‐interconnected ionomer network, conducive to the proton conductivity and more exposures of three‐phase sites. Then we calculated the radial density distributions illustrating the interaction between the SO_3_^2−^ and Pt nanoparticles (noted as Pt‐S, Figure S19a, Supporting Information), and between the whole side chain and Pt nanoparticles (noted as Pt‐SC, Figure 8c). As seen that both systems show similar radial distribution characteristics in the atomic density profiles of SO_3_^2−^/side chain around the Pt atoms. Three peaks are roughly identified in the curves. The first peak locates at around 3 Å, suggesting that the side‐chain groups are adsorbed by Pt atoms at an average distance of around 3 Å. In terms of the density values, the side chain of SSC‐PFSA shows lower adsorption ability to Pt atoms than that of LSC‐PFSA, which agrees with the above flexibility estimation. Similarly, the Pt‐S atom density of SSC‐MEA is also lower than that of LSC‐MEA (Figure S19a, Supporting Information). Additionally, we compared the Pt‐O_2_ intensity of the first peak, demonstrating a decreased atom density in SSC‐MEA, which reveals the easier oxygen access to the Pt surface at 70% RH (Figure 8d). The radial density distributions illustrating the interaction between the side chain and O_2_ (noted as SC‐O_2_) are depicted in Figure S19b (Supporting Information), suggesting the long side chain can adsorb the O_2_ strongly, hindering its diffusion through the ionomer assemblies and its arrival at the Pt active sites. To characterize the oxygen transport coefficiency in both systems, the self‐diffusion coefficient was computed. Figure S20 (Supporting Information) presents the self‐diffusion coefficient and solubility parameter of oxygen in both MD systems. As is seen, the LSC‐MEA system shows a lower diffusion coefficient of oxygen gas but a larger solubility parameter of oxygen gas than the SSC‐MEA system. This indicates that the long side groups in the LSC‐MEA system enhance the resistance of oxygen transport and possess stronger interaction with oxygen gas. These results demonstrated that the SSC‐MEA system is equipped with the desired interaction (including van der Waals forces and hydrogen bonding) and a well‐connected ionomer network, contributing to the enhanced cell performance.

## 3 Conclusion

This study delved into a comprehensive comparison between the two MEAs bonded by LSC‐PFSA and SSC‐PFSA ionomers, unraveling the intricate impact of the side‐chain on the interfacial interactions among Pt/ionomer/C. Such interactions behave distinct cathodic structures, with SSC‐PFSA fibrils forming thinner thickness, fostering a hierarchical‐porous loose structure, and supporting a more uniform dispersion of Pt. Adversely, LSC‐MEA exhibits a more compact structure with thicker ionomer fibrils, harmful to mass transport. In this context, this paper aims to fabricate a clear relationship of microstructure‐transport‐performance in both LSC‐MEA and SSC‐MEA. Detailed performance tests, containing polarization curves and EIS analyses at various humidities, indicate decreased voltage‐drop in SSC‐MEA, ascribed to lower total resistances. Thus, quantifying the respective contributions in the transport processes can clarify transport mechanisms and validate the pivotal role of superior mass transport in achieving enhanced fuel cell performance. O_2_ transport in the cathode, from the GDL through the pores and subsequent ionomer/carbon agglomerates, culminating in the Pt active sites, is intricately tied to cathodic structural characteristics. Through meticulous morphological characterization, mass‐transport quantifying, and performance tests, we comparatively analyzed the two CLs’ structures visually, ultimately verifying the SSC‐MEA as an exemplary transport‐friendly structure for several reasons:

1. Pore hierarchy advantage of SSC‐MEA. More primary pores (<20 nm), proper secondary pores, and more large pores (> 80 nm) in the loose cathode signify terrific O_2_ diffusion and water‐draining management.
2. Thinner SSC‐PFSA ionomer fibrils, reinforced by “ionomer bridges”, assemble into continuous proton‐conductive networks, favoring both proton and O_2_ transports.
3. Expansion of inter‐chain spacing in SSC‐PFSA with a higher water‐uptake capacity at humidified condition is favorable for O_2_ diffusion through the ionomer fibrils.
4. Molecular dynamics simulations confirm weaker bonding between short‐side‐chain and Pt, and stronger interaction between O_2_ and Pt, suggesting desired interaction in the SSC‐MEA system.

In conclusion, this paper provides profound insights into the mass transport mechanisms in the cathode, establishing a clear linkage of morphology‐transport‐performance. Such understanding significantly contributes to emphasizing the influence of binders on the cell performance.

## Conflict of Interest

The authors declare no conflict of interest.

## Supporting information

Supporting Information

## Data Availability

The data that support the findings of this study are available in the supplementary material of this article.
