# Supplementary material for: Transport‐Friendly Microstructure in SSC‐MEA: Unveiling the SSC Ionomer‐Based Membrane Electrode Assemblies for Enhanced Fuel Cell Performance
Source: Adv Sci (Weinh). 2024 Aug 15;11(39):2403647. doi: 10.1002/advs.202403647 (PMC11496990; doi:10.1002/advs.202403647)
Supplement: Supplementary file 1 — Supporting Information [file ADVS-11-2403647-s001.docx]

Supplementary Information

Transport-Friendly Microstructure in SSC-MEA: Unveiling the SSC Ionomer-Based Membrane Electrode Assemblies for Enhanced Fuel Cell Performance

*Min Li^1^, Han Ding^2^, Jingnan Song^1^,* *Bonan Hao^1^, Rui Zeng^1^, Zhenyu Li^1^, Xuefei Wu^3^, Zachary Fink^3^, Libo, Zhou^1^, Thomas P. Russel^3^, Feng Liu^1*^ and Yongming Zhang^1*^*

1. Dr. M. Li, Prof. F. Liu, Prof. Y. Zhang

School of Chemistry and Chemical Engineering

Frontiers Science Center for Transformative Molecules

Center of Hydrogen Science

Shanghai Key Lab of Electrical Insulation & Thermal Aging

Shanghai Jiao Tong University

Shanghai, 200240, China

Corresponding author E-mail: fengliu82@sjtu.edu.cn; ymzhang@sjtu.edu.cn

1. Dr. H. Ding

School of Energy Power and Mechanical Engineering, North China Electric Power University, 102206, Beijing, China.

1. Dr. X. Wu, Dr. Z. Fink, Prof. T. P. Russel

Materials Sciences Division, Lawrence Berkeley National Laboratory, Berkeley, CA 94720, USA.

Experiments

1. Materials

The 10.5 % wt. ionomer (LSC-PFSA and SSC-PFSA) solutions were provided by Dongyue Shenzhou New Materials Co. Ltd., China, without further treatment. The IECs of the Nafion ionomer and SSC ionomer are 1.1 mmol/g (equivalent weight of 909 g/mol) and 1.4 mmol/g (equivalent weight of 714 g/mol), respectively.^[1]^ The model of gas diffusion layer (GDL) was JNT 20, which was supplied by JNTG company from South Korea. The 40% wt. Pt/C catalyst (JM) was supplied by Johnson Matthey company from America. n-propanol (99.9%) and ethanol were purchased from Sigma-Aldrich Chemical Reagent Co., Ltd.. Perfluorosulfonic acid proton exchange membrane was provided by Dongyue Shenzhou New Materials. The anode was supplied by Shanghai Maxim Fuel Cell Technology Co.,Ltd. The water used in this paper is deionized (DI) water.

1. Materials characterization
2. The samples were prepared by embedding process and frozen microtomy using Leica EM UC7/FC7 cryo-ultra-microtome from Leica Camera Inc. of Germany. The samples were locating in liquid nitrogen during slicing process.
3. Atomic Force Microscope (AFM) analyses were performed on an AFM FastScan Bio from Bruker using Multi75E-G probes with the Pt overall coating. The sample was located at a cigar humidor with a 69% humidity control bag for 48 h to control the humidity of ~70% before the measurement.
4. The transmission electron microscopy (TEM) images and scanning transmission electron microscopy (STEM) mappings were acquired by using FEI Talos F200X TEM from Thermofisher containing an accelerating voltage of 200 kV. The morphology and HRTEM images were obtained on the TEM microscopes. Energy-dispersive X-ray spectroscopy (EDS) and high-angle annular dark-field STEM (HAADF-STEM) were taken on the same STEM microscopes.
5. Field-emission scanning electron microscopy (SEM) was operated on the Sirion 200 from Field Emission, Inc. (FEI), US.
6. Specific surface area and porosity analyses were characterized by ASAP 2460 from micromeritics instruments of America.
7. Grazing-incidence wide-angle X-ray scattering (GIWAXS) and grazing-incidence small-angle X-ray scattering (GISAXS) were performed at Beamline 7.3.3, Advanced Light Source, Lawrence Berkeley National Laboratory. The incidence angle is 0.16°, and the beam energy is 10 keV. The charge-coupled device (CCD) distance is ~284 mm for in GIWAXS and ~ 3578 mm for in GISAXS; thus, the q range of 0.5 to 3.4 Å^−1^ and 0.004 to 0.2 Å^−1^are obtained, respectively. Samples were prepared on the Si substrates using spin-coating method.
8. MEA preparation

A diagram of the MEA process routing was shown in Figure S1. The cathode was prepared from an ink containing 0.528 g of catalyst (40% Pt/ Vulcan XC-72R, Johnson Matthey), 2.113 g of PFSA ionomer solution, and 2.359 g of solvents (20% wt. NPA in DI water). The ratio of ionomer to carbon is set as 0.7. The cathodic catalyst layer with a Pt loading of 0.35 mg cm^−2^ was fabricated using OSP (OSG System Products Co., LTD) bar coaters to coat on PTFE substrate (supplied by Shanghai Maxim Fuel Cell Co., Ltd., China) at 50 ℃ first. The as-prepared cathode and anode were then transferred printed (1.0 MPa, 150°C for 105 s) onto PEM to make the catalyst-coated membranes (CCMs). The anode is supplied by Shanghai Maxim Fuel Cell Co., Ltd. With a Pt loading of 0.1 mg/cm^2^. The MEAs were finally prepared by hot-pressing the CCM with two sheets of GDLs (1.0 MPa, 125°C for 105 s).

1. Rheological analysis:

A rheological study of the ink was performed on a rotational rheometer (Discovery HR-20, TA instruments) using a parallel-plate geometry with a diameter of 40 mm and 1 mm gap at 25 °C. The ink tests including flow curve, three interval thixotropy test (3ITT), amplitude sweep**,** and frequency sweep. The shear rate range of the flow curve was from 0.1 to 500 s^−1^. In the flow curve test, the shear-thinning behavior with shear viscosity (η) declining escalating alongside shear rate was describe by a power-law correlation (Eq 1). The specific value of n can be deduced from the slope of the line in the graph,

$\eta=\frac{\sigma}{\dot{\gamma}}=\kappa\dot{\gamma}^{n-1}$ Eq 1

where where σ is the shear stress; κ is the fluid consistency coefficient; $\dot{\gamma}$ is the shear rate; and η is the measured viscosity, n is the shear-thinning index. The thixotropy test was performed in a three-stage scheme. First, the catalyst inks were sheared at a low shear rate ($\dot{\gamma}$) of 1 s^−1^ for 50 s, then at a high shear rate of 100 s^−1^ for 50 s, and eventually at a low shear rate of 1 s^−1^ for 200 s. For the amplitude sweep test, the angular frequency was a constant scan angle frequency (ω) of 1 rad s^−1^. For the amplitude sweep, the oscillation strain amplitude ranged from 0.01 % to 500 % to obtain the storage modulus (G′) and the loss modulus (G″). For the frequency sweep, the critical strain and linear viscoelastic region (LVR) where ink can flow were determined by the amplitude scan. Then, the frequency scan was performed in a range smaller than the critical strain, with a strain amplitude of 0.1% and ω in the range of 10^−1^ −100 rad s^−1^. In the amplitude sweep test, the yield stress σ_y_ and critical strain amplitude γ_c_ can be determined from the maximum in the loss modulus G'' for the systems with weak strain overshot of G'' similar with the ink used in this study.^[2]^

1. Polarization curves

The assembled single cells with an effective area of 25 cm^2^ were tested on a 100 W FC test station (850Es, Hephas Energy Co., Ltd., Taiwan). The cell was held for 3 hours with a voltage of 2 A cm^−2^ before the polarization curves were recorded. The performance test conditions were: cell temperature of 65 °C, the gas flow rate of 2 standard liters per minute (SLPM) and 5 SLPM for pure H_2_ and air, respectively, and back pressures both of 150 kPa (250 kPa abs). The polarization curves were recorded in current-control mode, from 0 to 2 A cm^−2^ and holding each potential for 3 min to reach a steady state. The polarization curves were obtained at five conditions: 65°C (100% RH), 65°C (70% RH), 65°C (50% RH), 65°C (30% RH), and 65°C (15% RH).

1. Electrochemical surface area analysis (ECSA)^[3]^

CV curves were obtained in a potential range of 0.08 V – 1.0 V vs. RHE at 65 °C and a scan rate of 50 mV s^−1^. The CV test conditions were: the gas flow rate of both 2 SLPM of pure H_2_ and 4 SLPM of N_2_, and the back pressures of 100 kPa. The ECSA value was determined by integrating the hydrogen (desorption) region from 0.1 V to 0.4 V vs. RHE. The final cycle of a set of three cycles was used for data analysis. The ECSA (m^2^/ g_Pt_) was calculated using the following formula:

$S_{ECSA}=\frac{Q (mC)}{0.21(mC\cdot cm-2)\times M_{\mathrm{Pt}}(mg)}$ Eq 4

where Q and M_Pt_ (mg) are the integrated charge and mass loading of Pt, respectively, and 0.21 (mC m^−2^) corresponds to a monolayer hydrogen adsorption charge on polycrystalline Pt.

1. Electrochemical impedance spectroscopy (EIS)

EIS was conducted after polarization curve collections by imposing an alternating current (AC) signal to the FC and the amplitude for the ac impedance was 5% of the direct current (DC) over a frequency range of 10 kHz to 0.1 Hz. The EIS curves were recorded at 1A cm^−2^ and 2A cm^−2^ under corresponding operating condition using hydrogen (2 SLPM) and air (5 SLPM) with backpressure of 150 kPa.

1. Hydrogen crossover current density

The H_2_ crossover current density was measured by LSV under constant 0.5 SLPM/1 SLPM of H_2_/N_2_ using a scan rate (2 mV/s) between 0.05 V and 0.6 V at 65 ℃ and 100 % RH. The back pressure conditions are 50 kPa and 50 kPa for anode and cathode, respectively, and 100 kPa and 50 kPa for anode and cathode, respectively.

1. The internal resistance

The internal resistance was obtained in a battery tester instruments (AT526) from anbai, China. The test conditions are: cell temperature of 65 ℃, humidity of 100 %, continuous current of 2 A cm^−2^.

1. O_2_ transport resistance^[4]^

The O_2_ transfer process in the cathode is a main limiting factor for the limiting current density, which is an essential parameter for evaluating the electrochemical power generation capacity of fuel cells. The assembled single cells with an effective area of 5 cm^2^. The O_2_ transport resistance was evaluated from limiting current measurements at 65°C under 1.5 SLPM of H_2_ and 3 SLPM O_2_/N_2_ (x_O2_ = 1 %). Scan from 0.4 V to 0.1 V and repeat for 3 times to determine the average current density. The back pressure is set as 0, 50, 100, 150, and 200 kPa. The humidity condition is set as 30 %, 70 % and 90 %.

$C_{O_{2}}=\frac{P-P_{H_{2}O}}{RT}\times x_{O_{2}}$ Eq 5

$R_{\mathrm{tot}} =\frac{C_{O_{2}}\times4\times F}{J_{L}}$ Eq 6

where J_L_ is the limiting current density (A m^−2^), C_O2_ is the oxygen concentration (mol cm^−3^), P is the absolute gas pressure (Pa), P_H2O_ is the water vapor pressure (Pa), x_O2_ is the oxygen mole fraction, R is the gas constant (8.314 J mol^−1^ K^−1^), T is the gas temperature (K), and F is Faraday's constant (96485 C mol^−1^). The saturated vapor pressure of water vapor at 65 ℃ is 25043 Pa. For this study, the limiting current was specified to be the maximum current that was obtained from 0.4 V to 0.1 V repeated for 3 times.

1. H_2_-N_2_ impendance^[5-6]^

Proton conduction resistance was evaluated by H_2_-N_2_ impedance on the condition of various humidities (RH 15 %, 30 %, 50 %, 70 %, 100 %) at 65 ℃. The H_2_-N_2_ impedance was conducted by imposing a bias voltage of 0.5 V to the cell and the amplitude for the AC impedance was 10 mV over a frequency range of 100 kHz to 0.1 Hz.

$Z=Z^{'}+Z^{''}=\frac{Z_{H^{+}}}{3}+\frac{1}{j\omega C} \omega\to0$ Eq 7

where Zis the total impedance, Z’ and Z’’ are the real and imaginary parts of impedance (Ω cm^2^), respectively. $Z_{H^{+}}$ is the proton resistance per geometric area of electrode in CL (Ω cm^2^). ω is the frequency (rad s^−1^). C is the double-layer capacitance per geometric area of electrode (F cm^−2^) in CL, j is the square root of -1.

1. Simulation details and methodology

In this work, classic molecular dynamics (MD) simulations are performed to gain the structural properties of Pt/C/polymer systems. The chemical structures of ionomers for simulation are shown in Figure S21. For Pt/C structure, two Pt clusters with 4 Pt atoms are placed onto a carbon nanoparticle composed of 192 carbon atoms. First, two MD simulation boxes with dimensions of around 85 × 85 × 85 Å^3^ are created. Then, polymer chains and Pt/C structures are randomly placed into as-created MD boxes, in which the mass ratio of polymers-to-carbon particle-to-Pt is set to be 0.7:1.0:2/3 that is consistent with the experimental characterizations. In detail, A and B systems are mainly composed of 56 Pt/C particles, and 16 polymer chains, 500 O_2_ and 185 H_2_O water molecules. Th Pt/C particle is structurally characterized by single-layer fullerene structure with 192 carbon atoms that are decorated by two Pt clusters on the surface, shown in Figure S22. In addition, water and O_2_ molecules are also inserted into as-created MD boxes to achieve humidity of 70 % conditions that are similar to our experimental settings. Such two mixture hybrid structures are generated by the Amorphous Cell module. Periodic boundary conditions (PBCs) are imposed in the three orthogonal directions to mimic large structural samples. To describe the atomic interactions in both systems, the COMPASS (condensed-phase optimized molecular potentials for atomistic simulation studies) forcefield^[7]^ is employed. For the non-bonded interactions in the system, the 9-6 Lennard-Jones potential with cutoff distance of 12.5 Å is applied to describe the van der Waals (vdW) forces between atoms, while the standard Coulomb potential is utilized to mimic the atomic electrostatic interactions that is evaluated by the particle−particle particle−mesh (PPPM) algorithm. Prior to MD simulations, geometry minimizations are firstly performed to relax both systems with energy and force tolerances of 0.001 Kcal/mol and 0.5 Kcal/(mol·Å), respectively. Finally, MD simulations with 2,000,000 timesteps are carried out to relax the systems under canonical (NPT) ensemble at temperature of 338 K and confining pressure of 0.25 MPa, in which the temperature and pressure are controlled by the Nose thermostat and Breendsen barostat techniques, respectively. The dynamics of atoms in the systems follow the classical Newton’s motion, in which the Velocity Verlet algorithm with timestep of 0.5 fs is applied to integrate the classic Newton’s equation.^[8]^ All the MD simulations are implemented using the Forcite Module of Materials Studio.


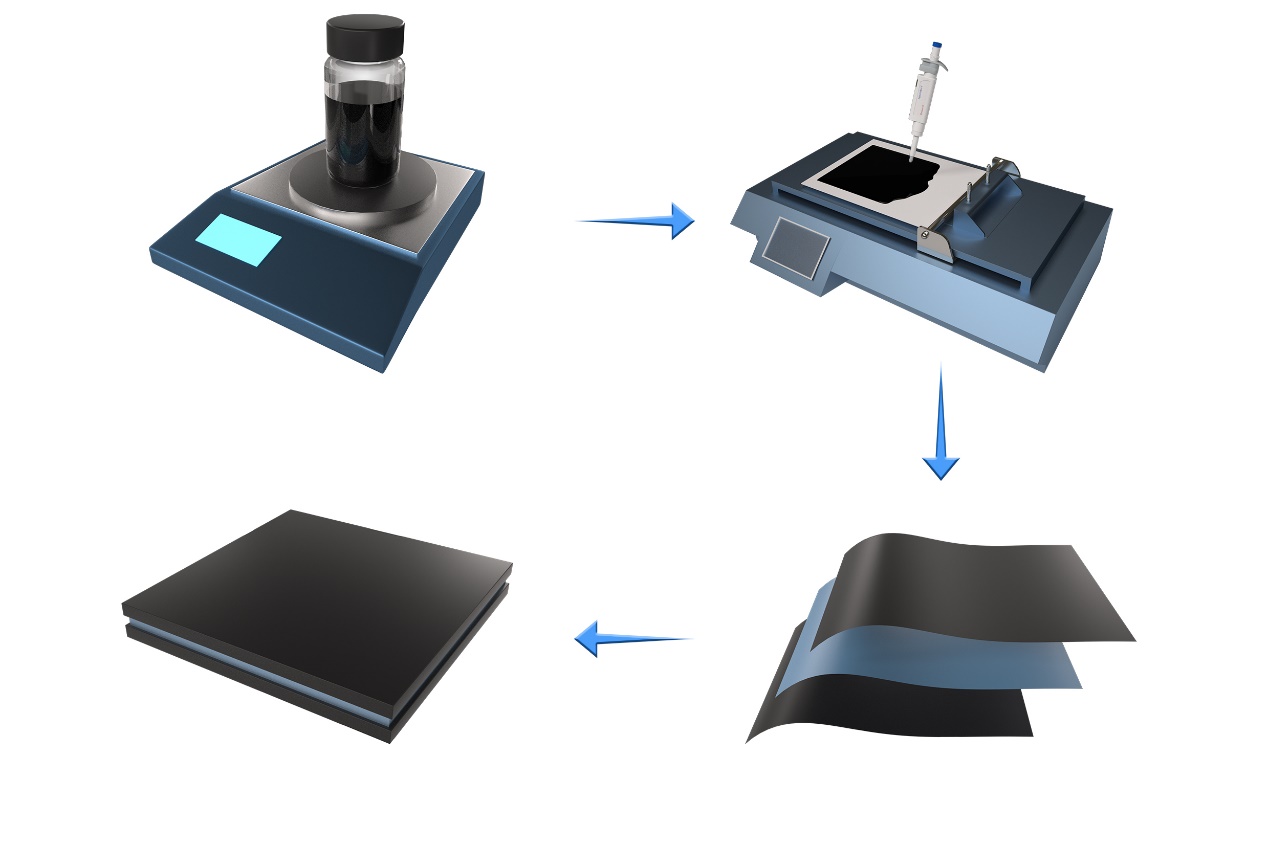


**Figure S1** scheme of the preparation of the MEA.


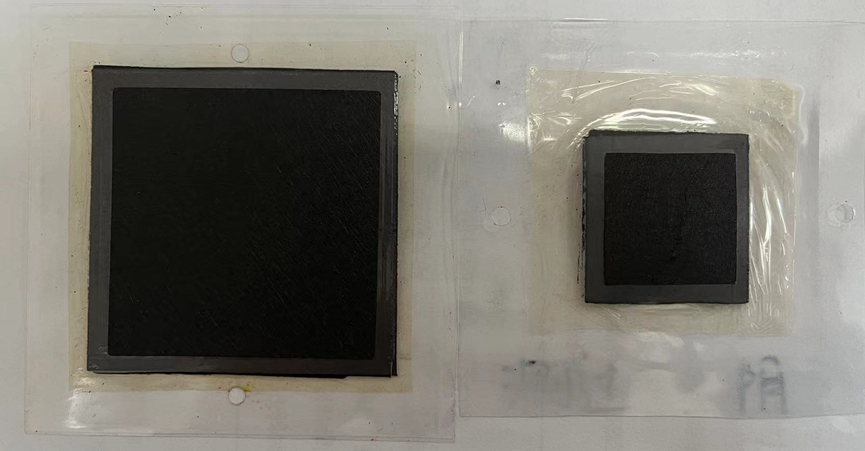


**Figure S2** The images of MEA with an effective area of 25 cm^2^ and 5 cm^2^.


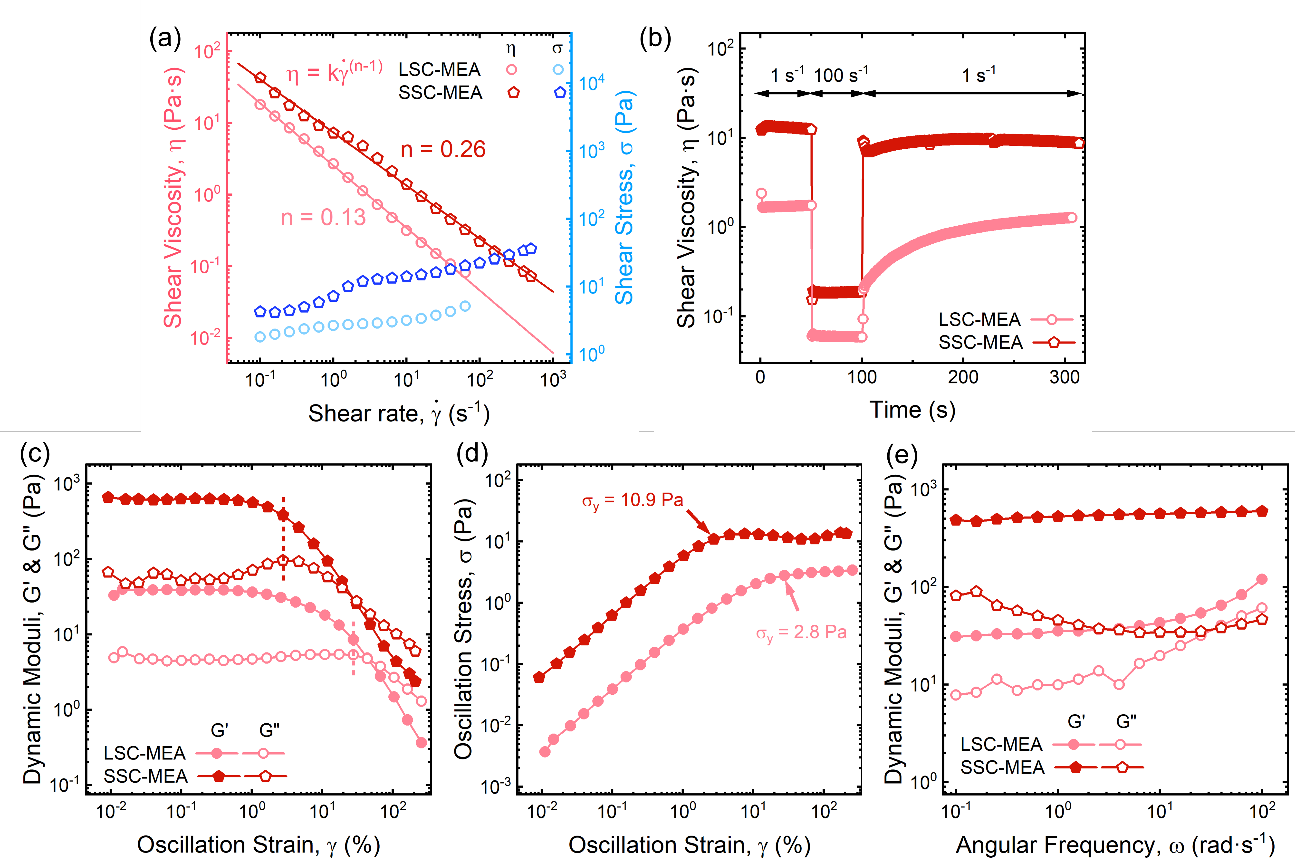


**Figure S3** Rheological analyses. (a) Steady-shear relative viscosities, (b) 3ITT analysis, (c) amplitude scan, (d) and as-calculated oscillation stress, (e) frequency scan of catalyst inks with different ionomer binders.


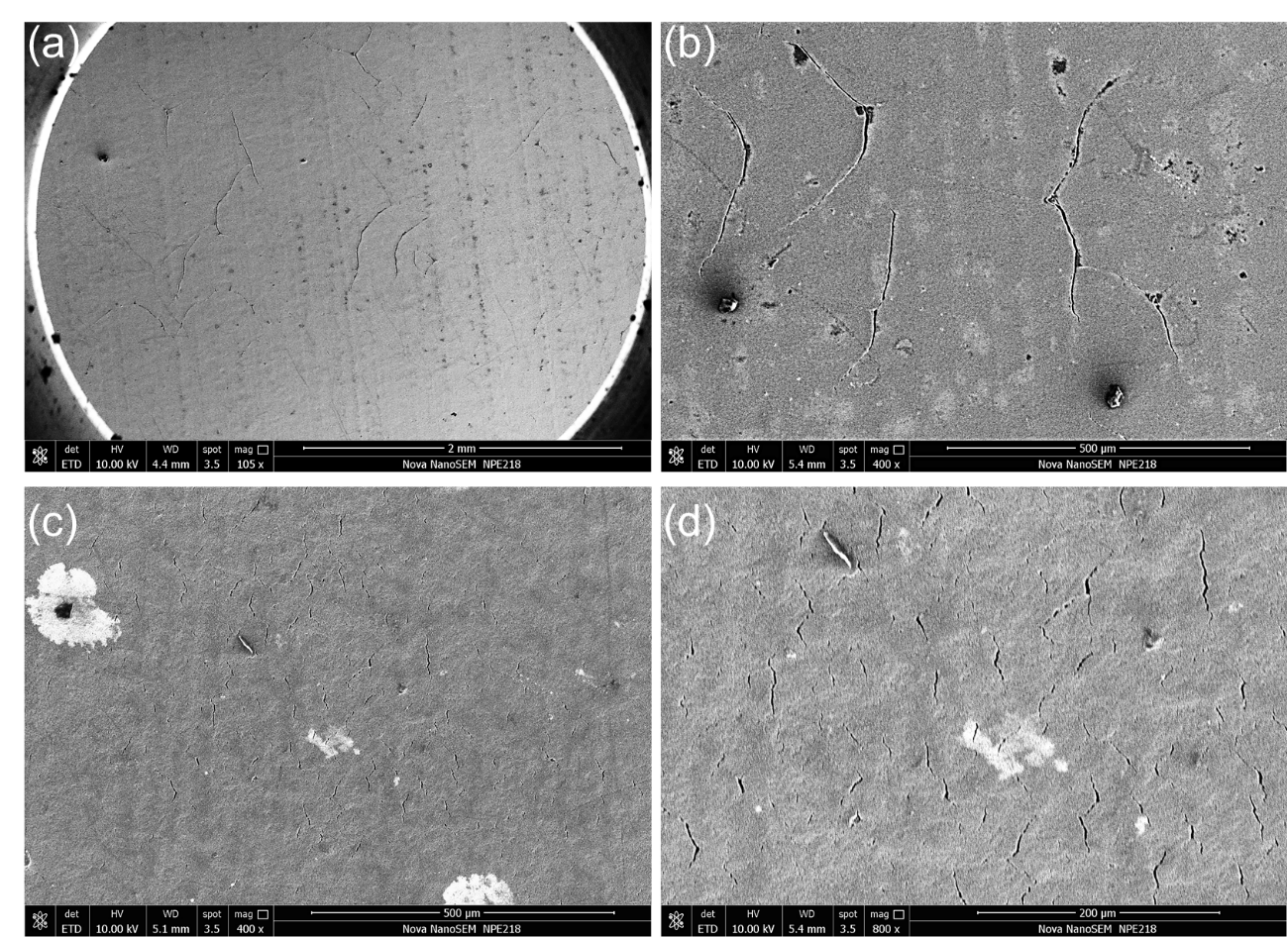


**Figure S4** SEM images of the coated cathode of (a-b) LSC-MEA and (c-d) SSC-MEA.


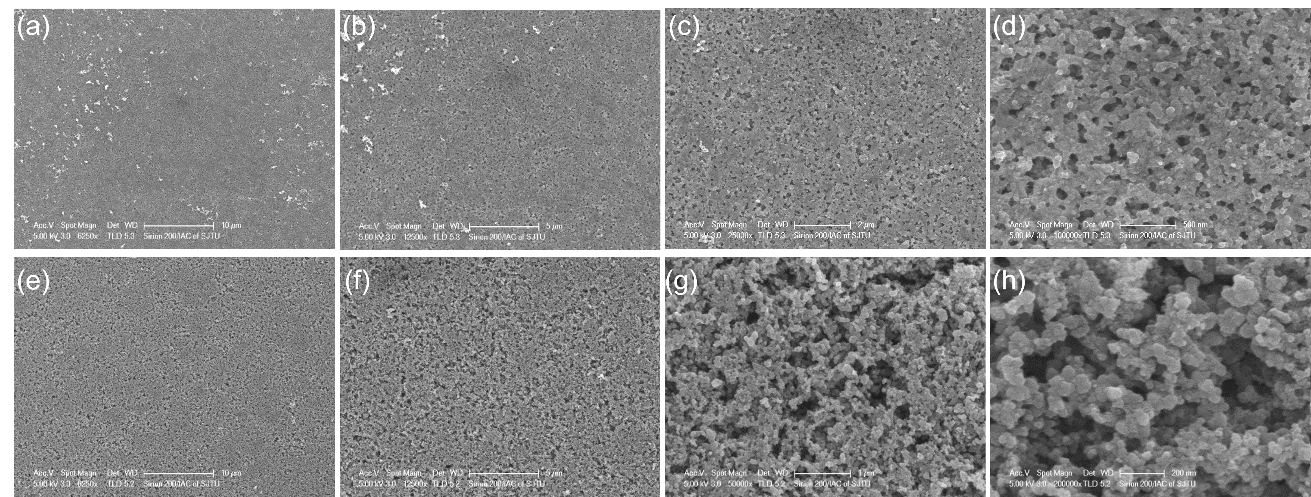


**Figure S5** SEM images of the surface morphology of the (a-d) MEA-LSC and (e-h) MEA-SSC.


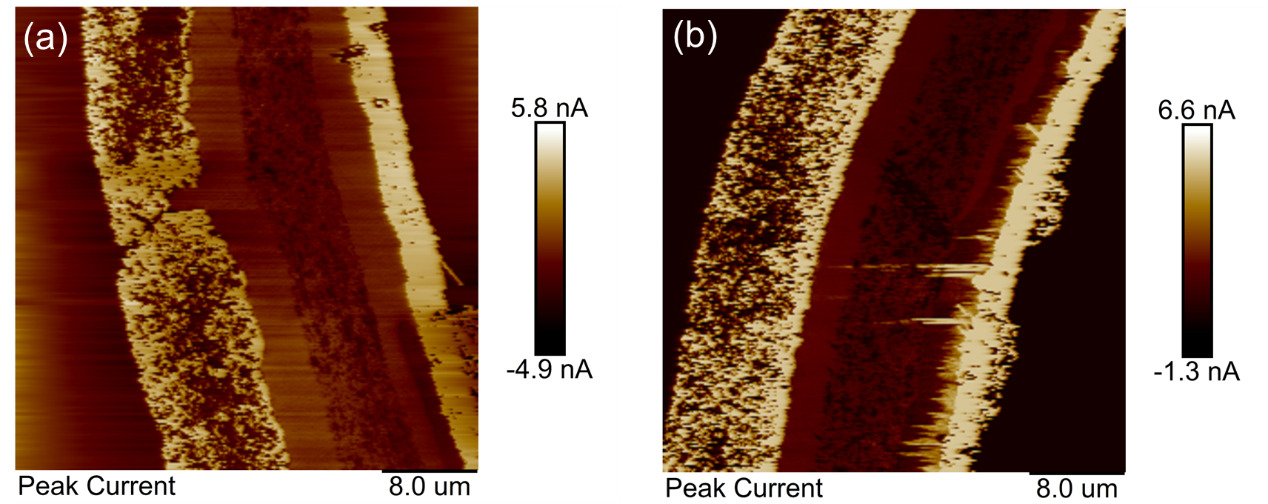


**Figure S6** AFM maps. The peak current image of (a) LSC-MEA and (b)SSC-MEA.


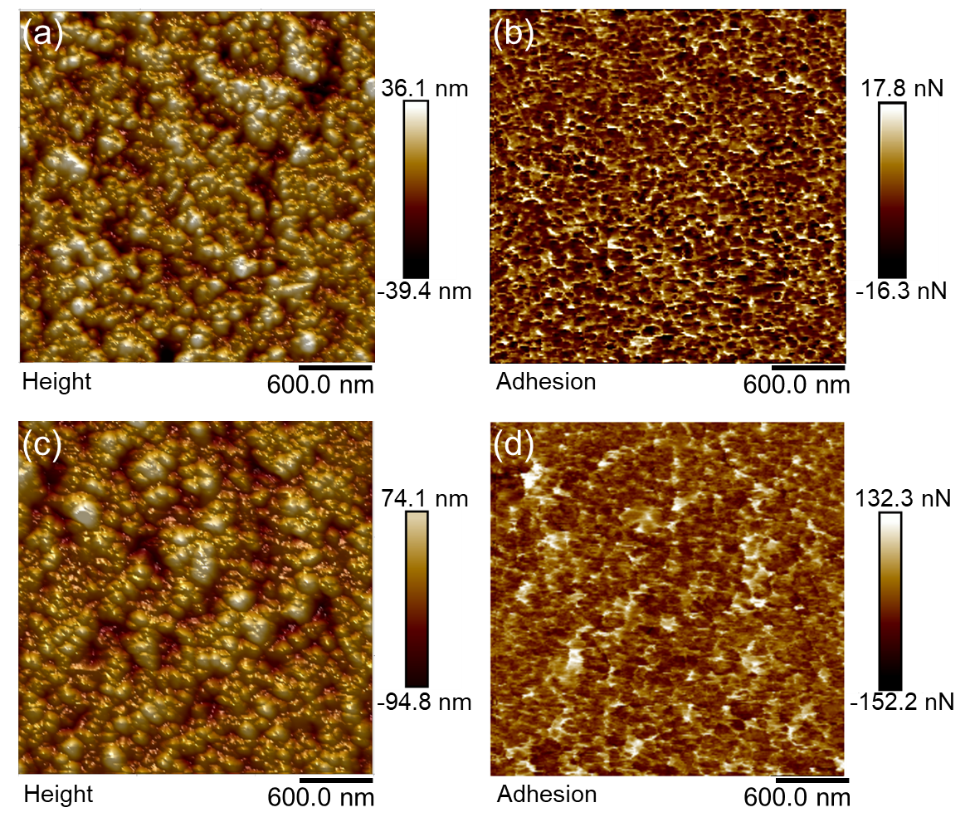


**Figure S7** AFM images of the cross-line morphology of the (a-b) LSC-MEA and (c-d) SSC-MEA.


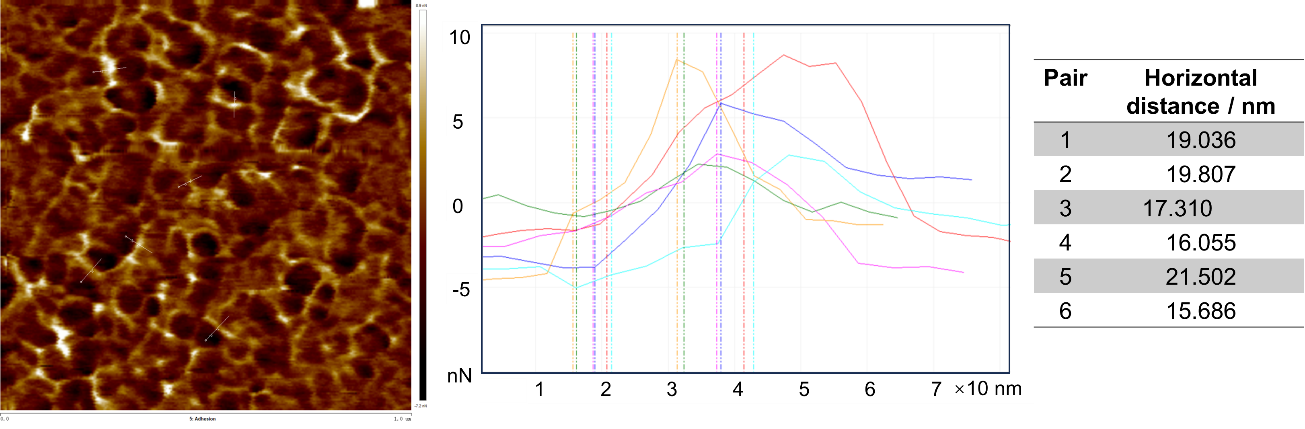


**Figure S8** The statistical average size of LSC-PFSA fibrils in the adhesion image.


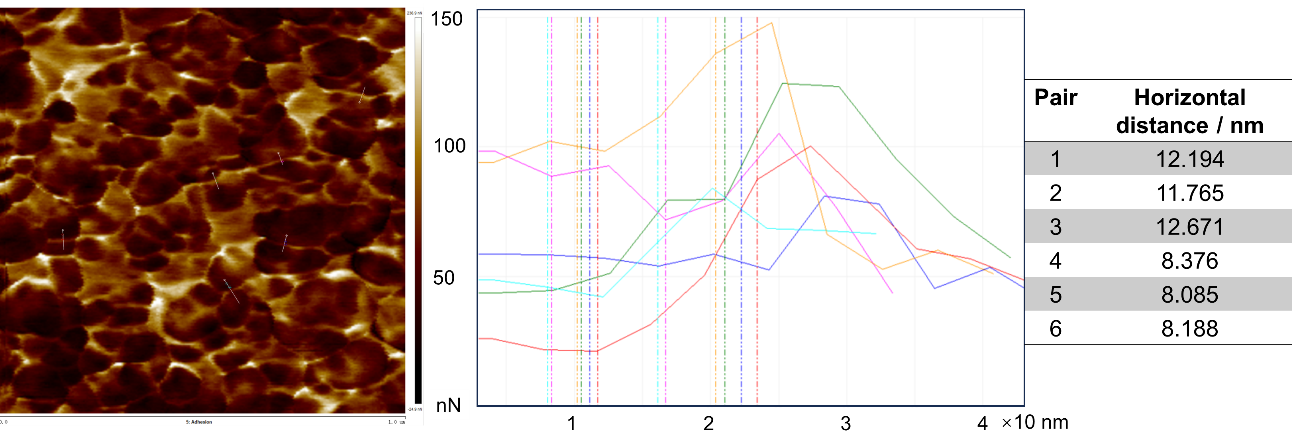


**Figure S9** The statistical average size of SSC-PFSA fibrils in the adhesion image.


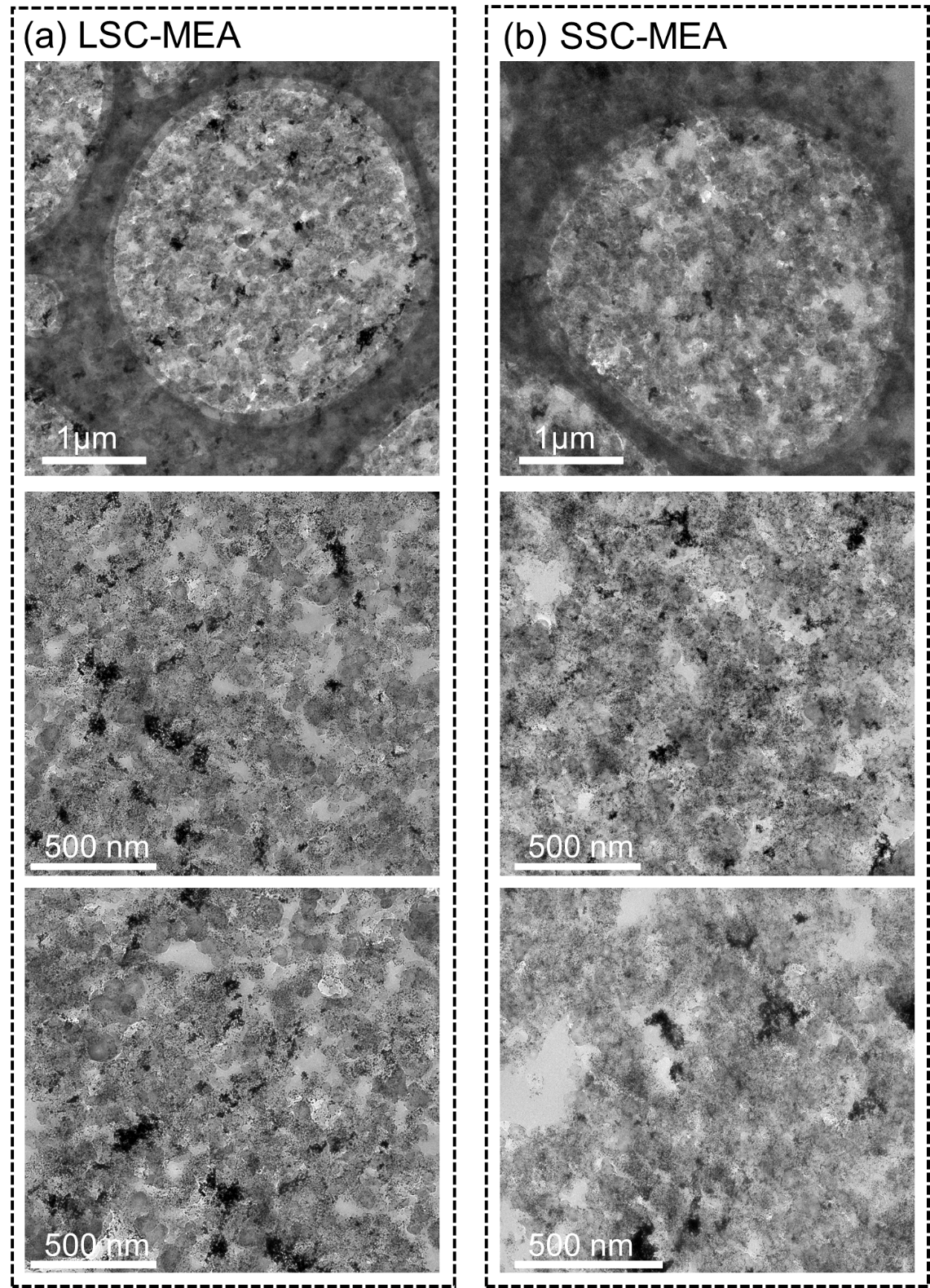


**Figure S10** TEM images of the (a) LSC-MEA and (b) SSC-MEA.


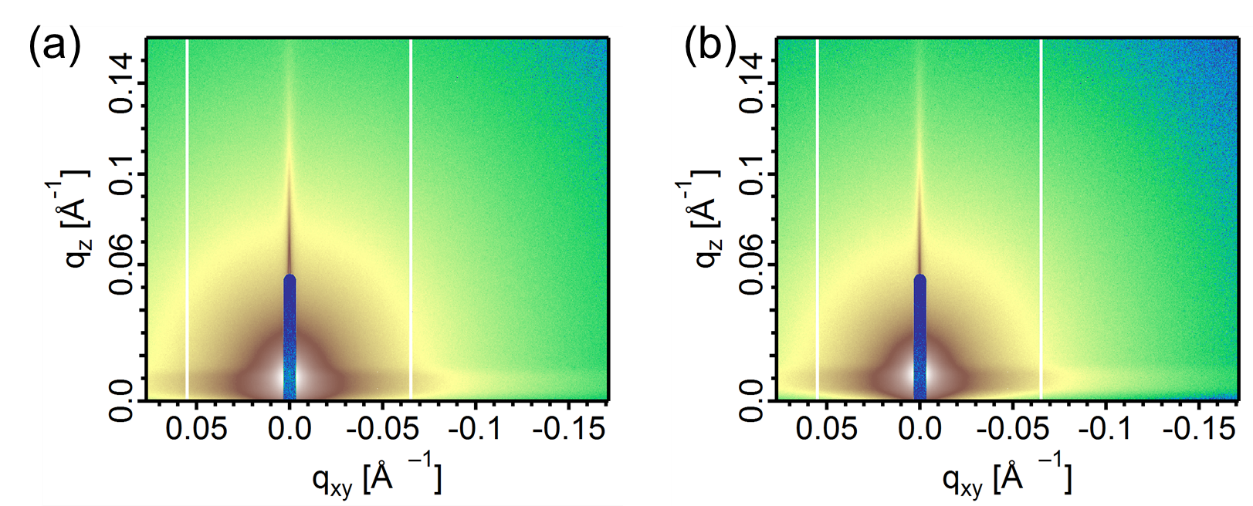


**Figure S11** 2D GISAXS patterns for (a) LSC-MEA and (b) SSC-MEA.


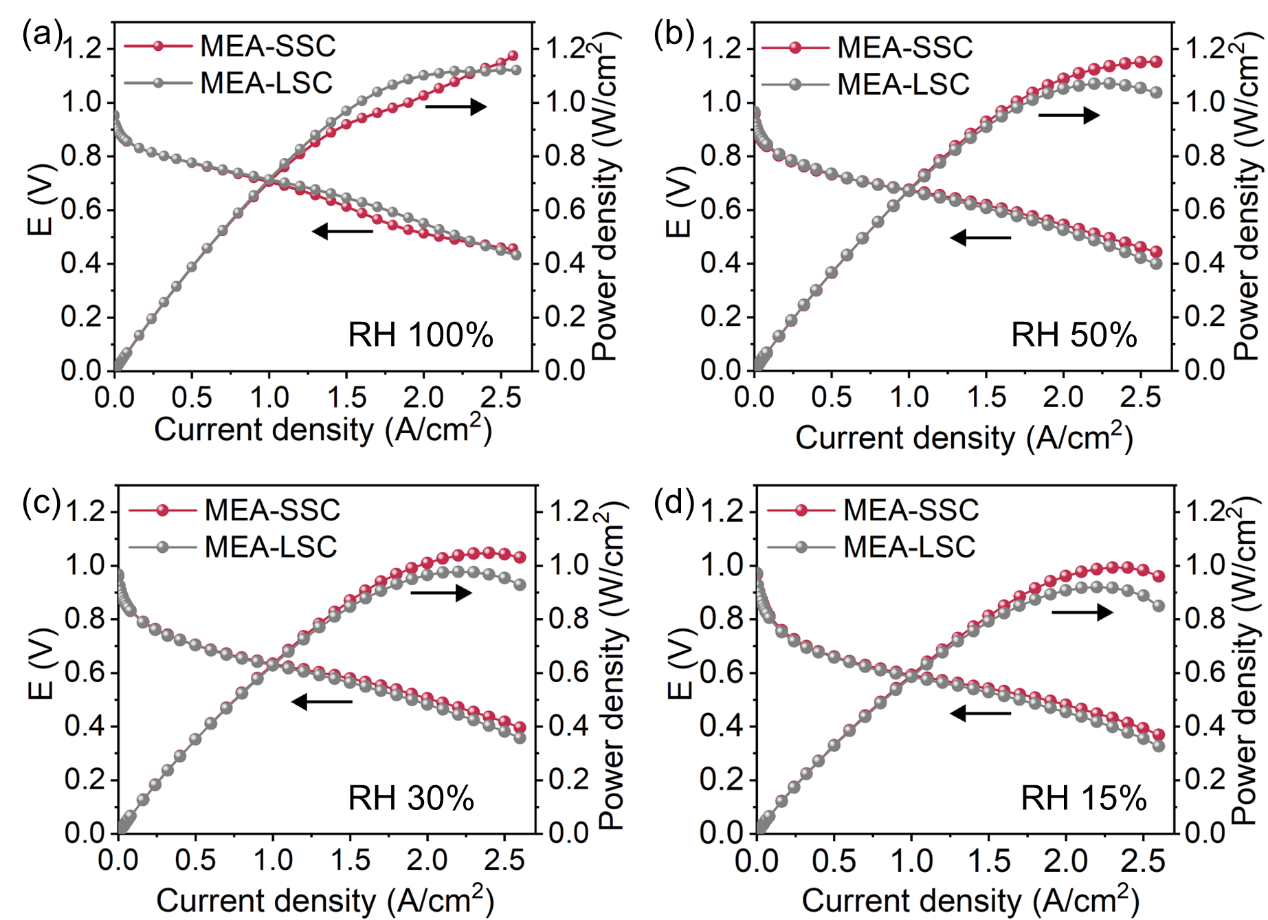


**Figure S12** Polarization curves at RH of (a) 100 %; (b)50 %; (c) 30% ;(d) 15%.

SSC-MEA also demonstrates higher power density at 2 A/cm^2^ at most humidities except at 100 % RH. LSC -MEA shows higher power density of 2 A/cm^2^ at RH 100%, which may be attributed to the water flooding occurred in the SSC-MEA. However, the SSC-MEA makes a comeback at 2.3 A/cm^2^, which may benefit from the superior pore structure, draining away surplus water.


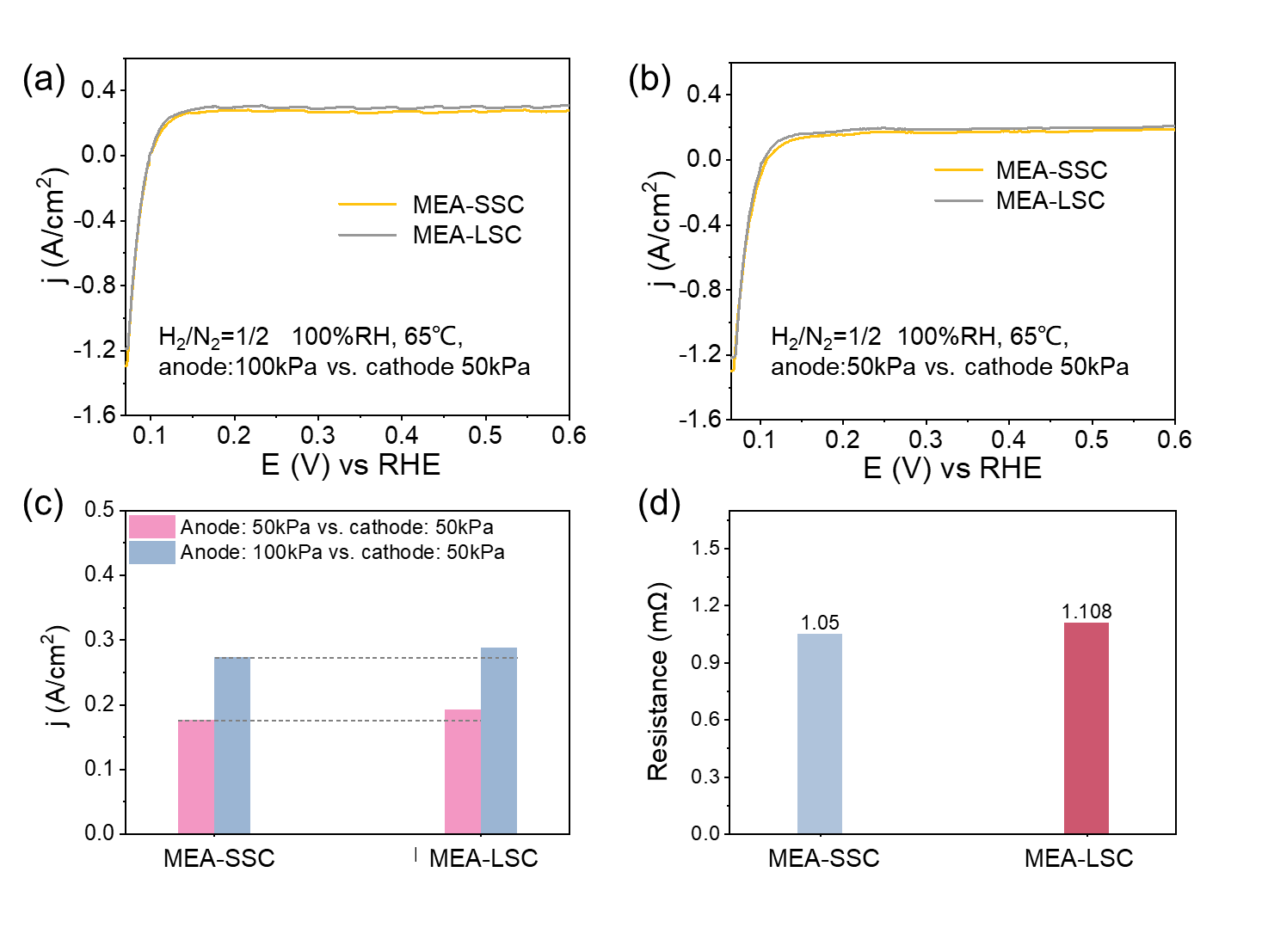


**Figure S13** Hydrogen penetration current density curves at (a) 100 kpa (anode), 50kpa (cathode) and (b) 50 kpa (anode), 50kpa (cathode); (c) the values of the hydrogen penetration current density at 0.4V; (d) the internal resistance of the single cell.


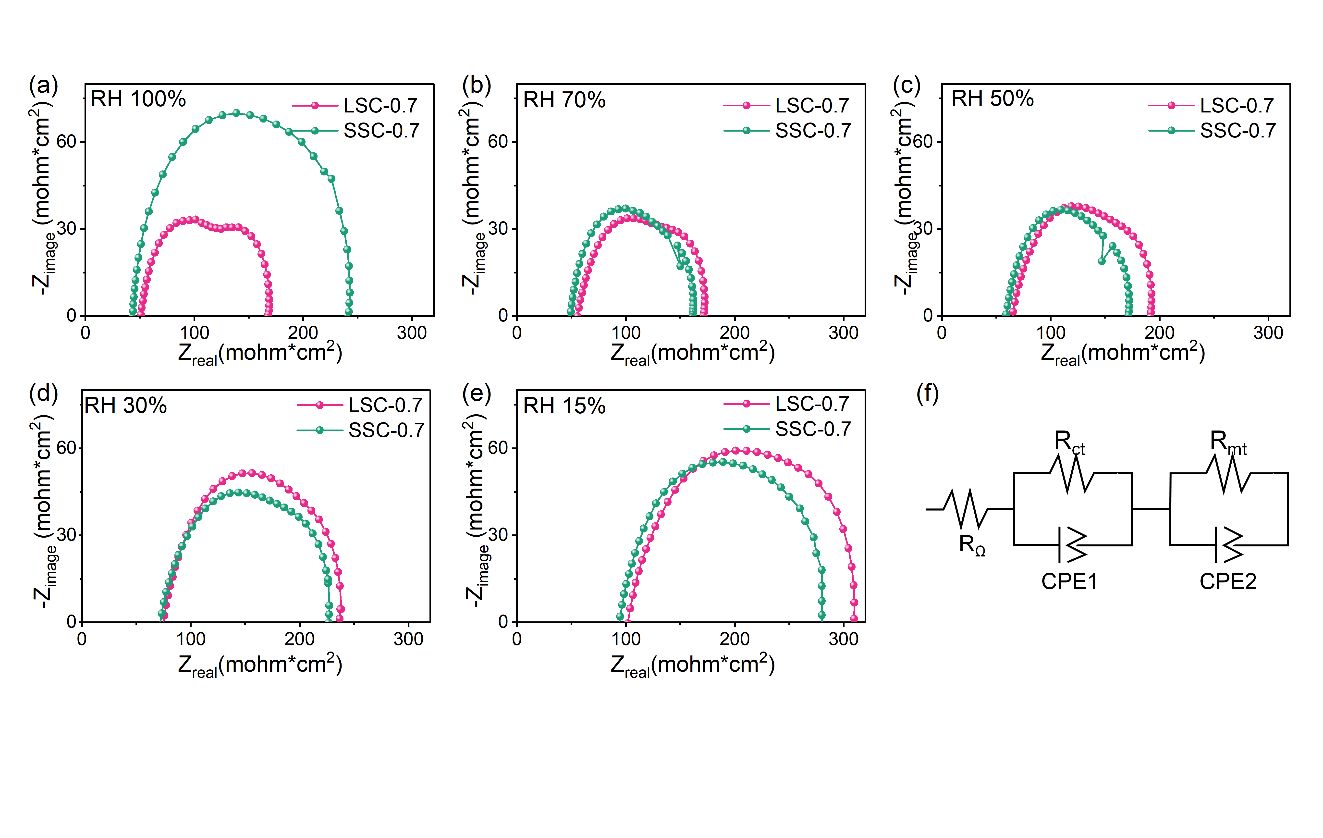


**Figure S14** The EIS spectra of the LSC-MEA and SSC-MEA for 1 A/cm^2^ at (a)100 RH; (b) 70 RH; (c) 50 RH; (d) 30 RH; (e) 15 RH and (f) their corresponding equivalent circuit diagram.


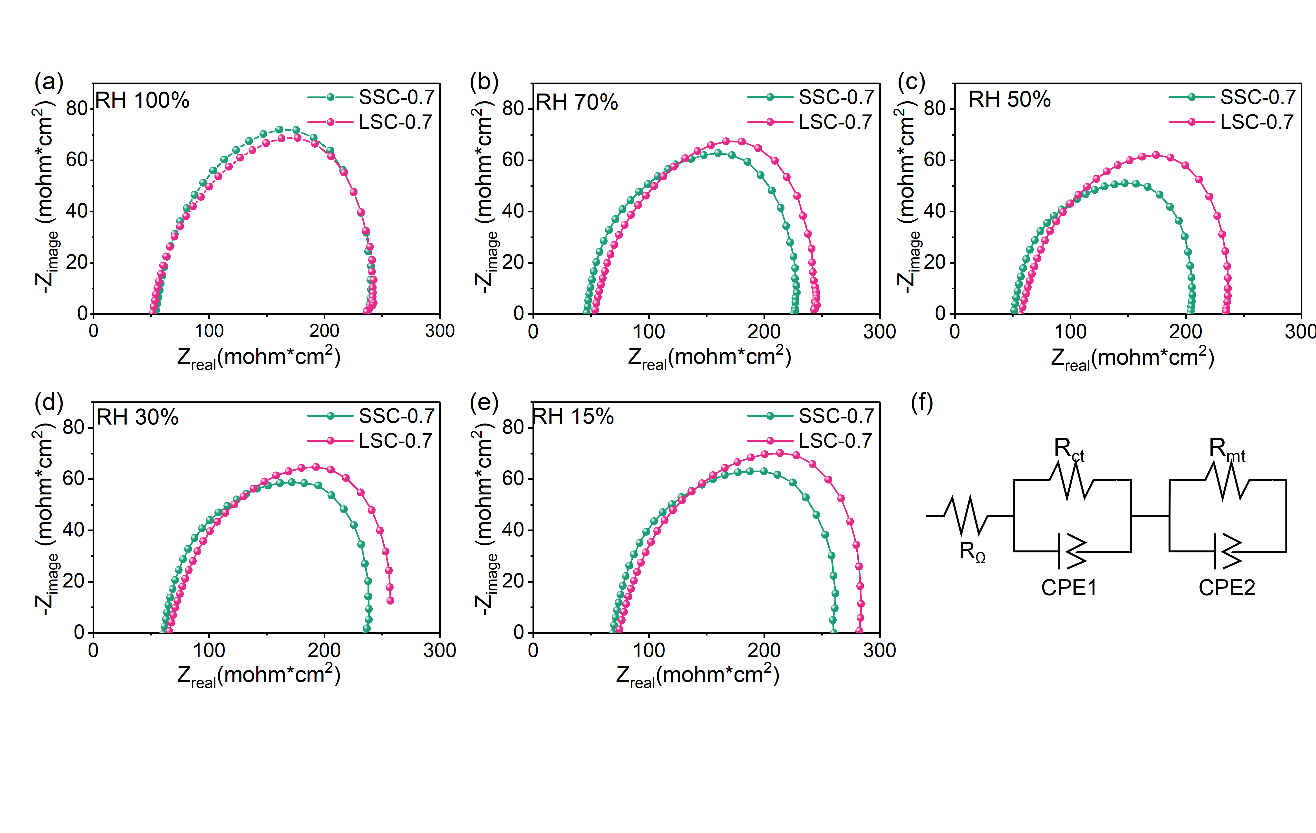


**Figure S15** The EIS spectra of the LSC-MEA and SSC-MEA for 2 A/cm^2^ at (a)100 RH; (b) 70 RH; (c) 50 RH; (d) 30 RH; (e) 15 RH and (f) their corresponding equivalent circuit diagram.


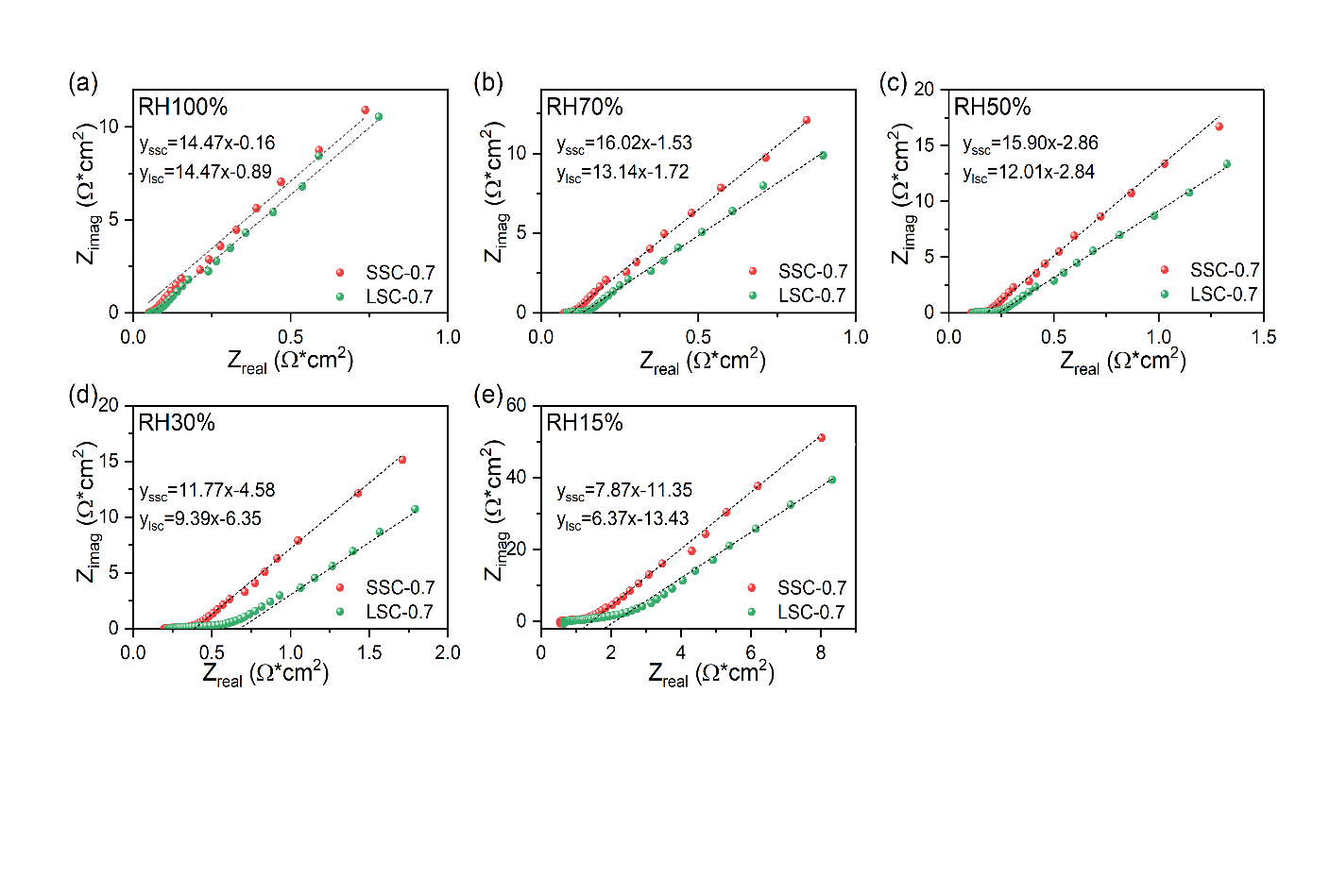


**Figure S16** The H_2_-N_2_ impedance spectra at various humidty of (a) 100 RH; (b) 70 RH; (c) 50 RH; (d) 30 RH; (e) 15 RH.

The curves were horizontally shifted to Zreal = 0, thereby mitigating the impact of Ohmic impedance. Construct a tangent line within the low-frequency domain and identify the point of intersection with the X-axis for the as-fitted line. The impedance at that intersection point corresponds to $\frac{Z_{H^{+}}}{3}$.


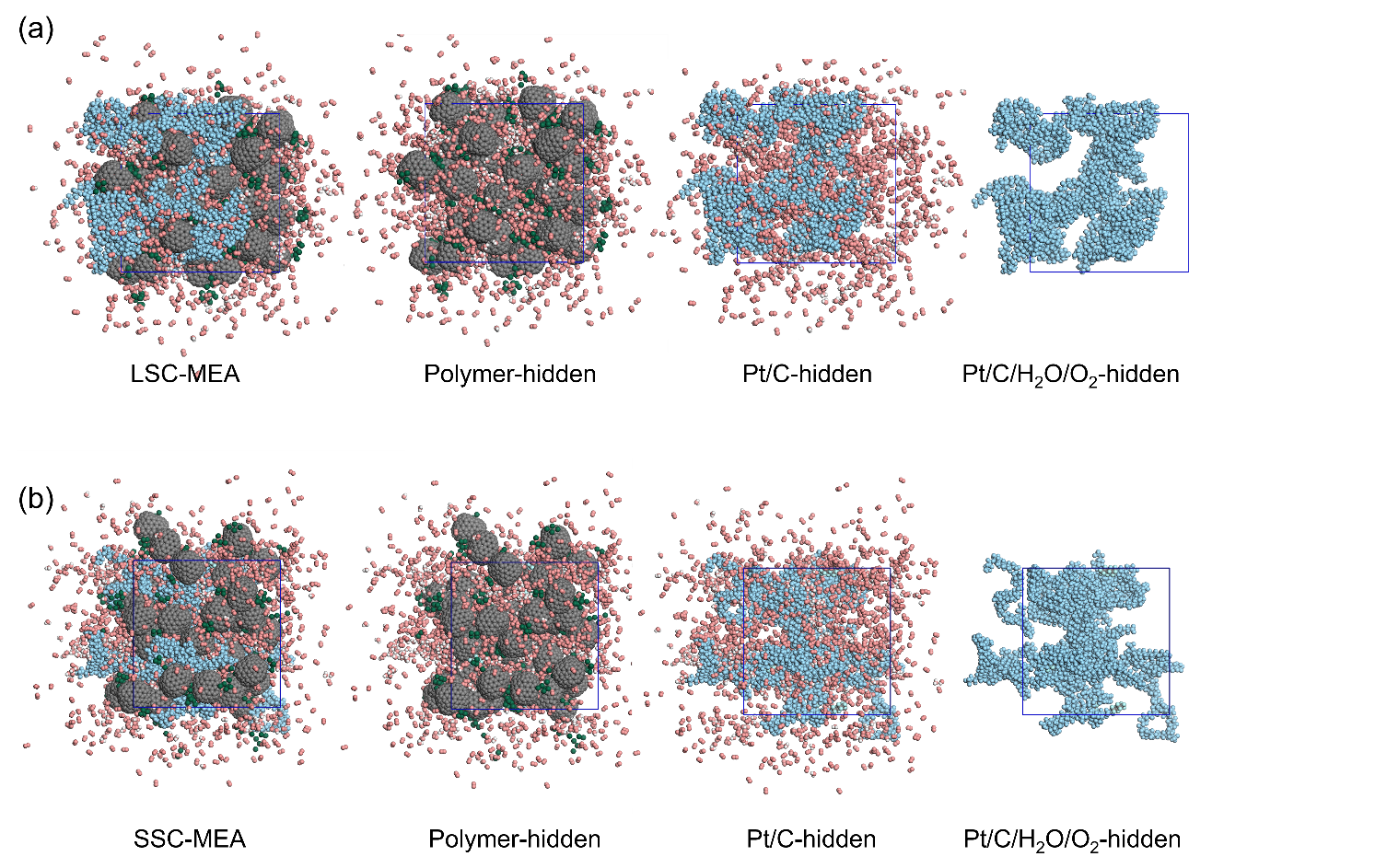


**Figure S17** Snapshots of configurations of (a) LSC-MEA and (b) SSC-MEA.


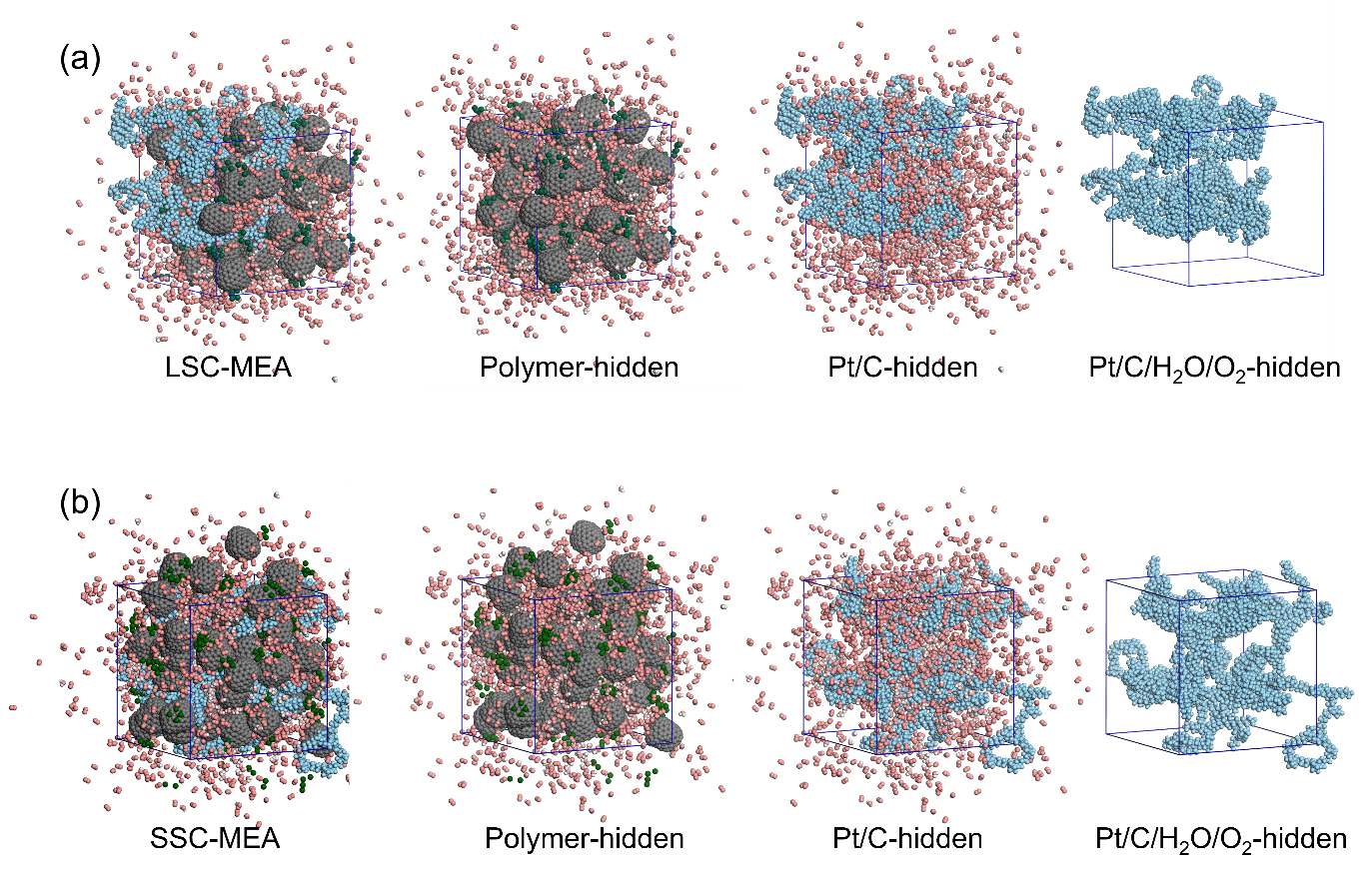


**Figure S18** Snapshots of the other view angle of (a) LSC-MEA and (b) SSC-MEA.


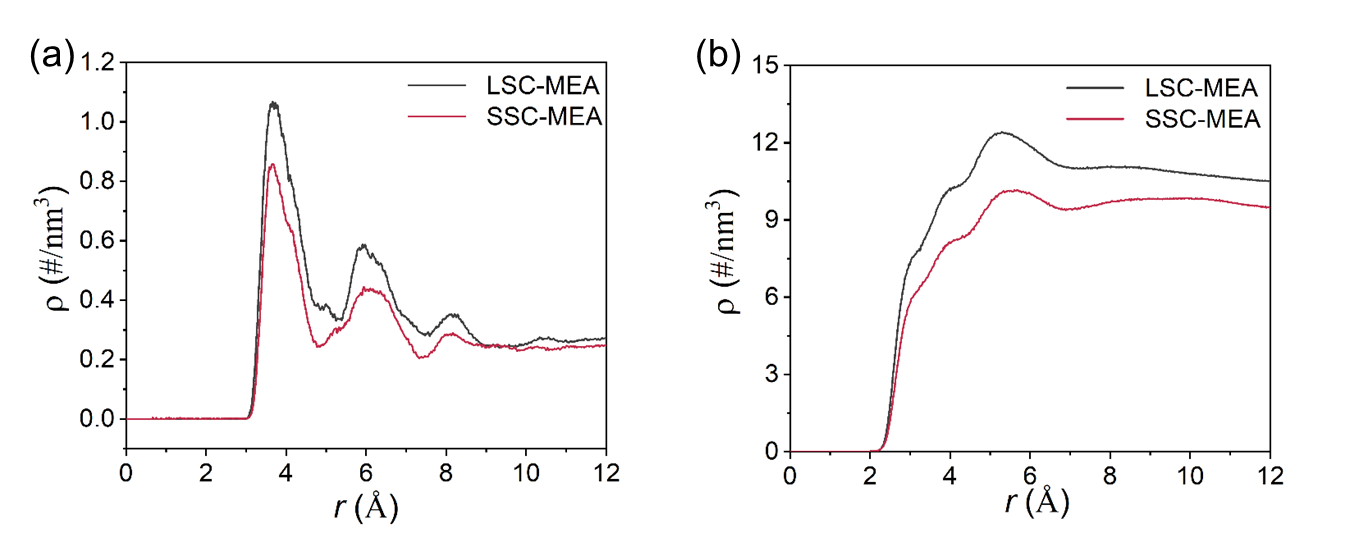


**Figure S19** The atomic density profile of the (a) Pt-S and (b) the side chain-O_2_.


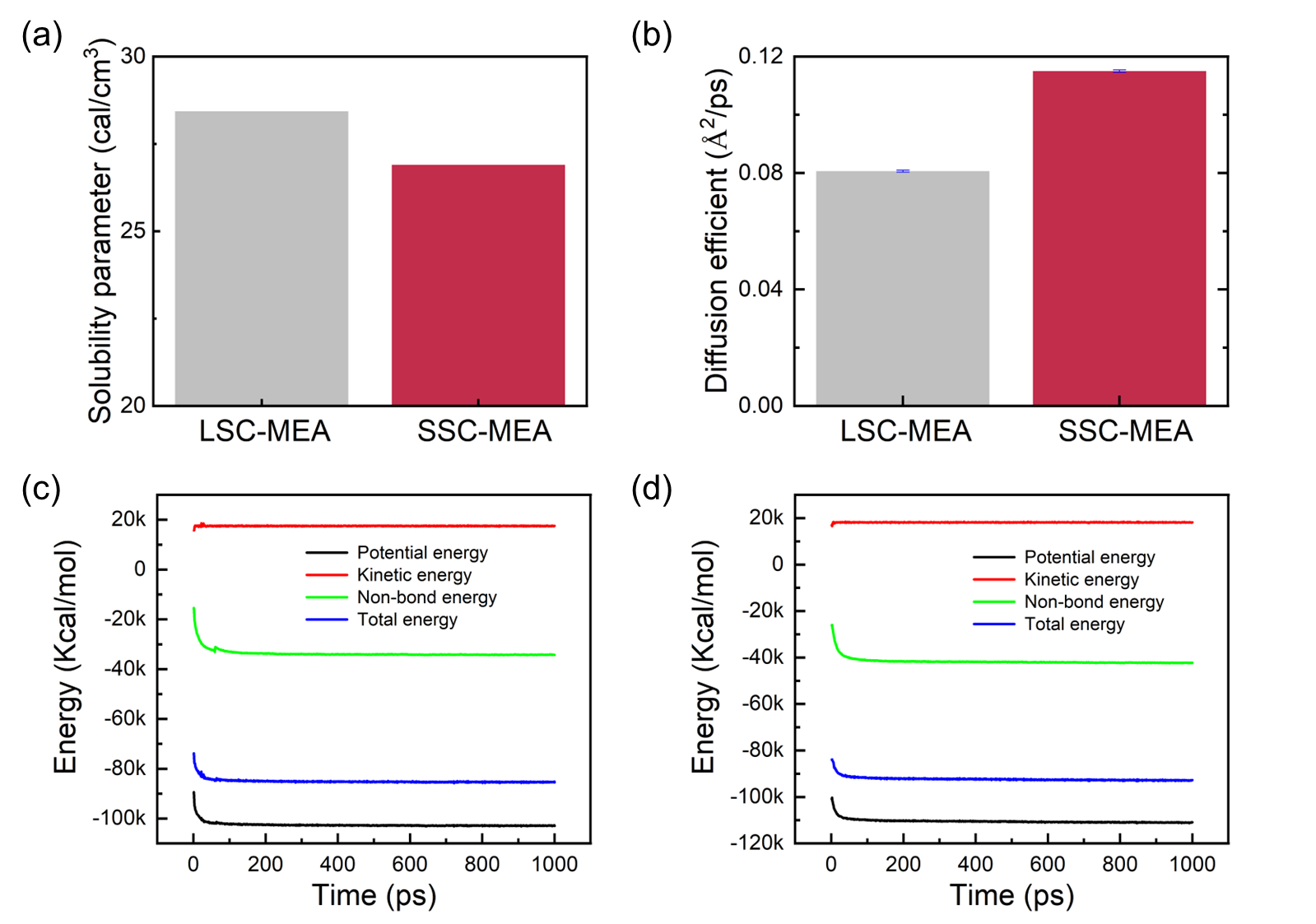


**Figure S20** (a) The O_2_ solubility parameter and (b) diffusion efficient of the LSC-MEA and SSC-MEA.

**Figure S21** Chemical structure of the simplified LSC-PFSA and SSC-PFSA for molecular dynamics simulations.


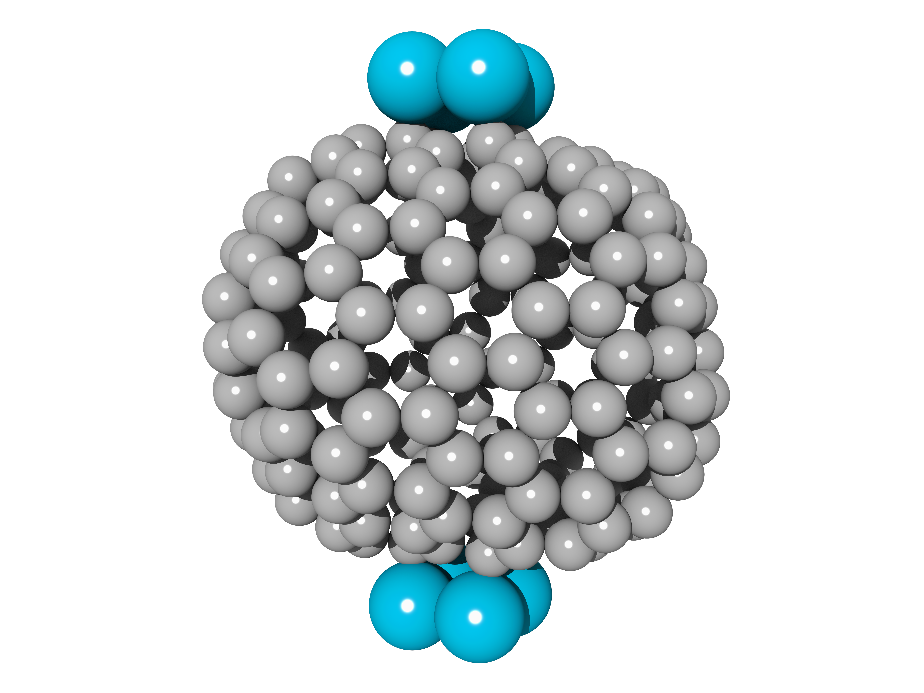


**Figure S22** MD model of Pt/C particle

| Ionomer | Ionomer wt. % or I/C | RH (%)  and cell Temperature (℃) | | Peak power density (W/cm^2^) | EIS Rtot | Cathode proton transport resistance  (mΩ cm^2^) | Paper |
| --- | --- | --- | --- | --- | --- | --- | --- |
| **Dongyue** | **0.7** | **65** | **70** | **1.23** | **0.16 at 1A/cm^2^** | **49.4** | **This paper** |
| Aquivion EW720 | 0.65 | 80 | 30 | 0.92 | 0.2 at 1A/cm^2^ | 18.6 | ^[9]^ |
| Aquivion, EW720 | 0.58 | 80 | 70 | 1.245 | 0.29 at 1A/cm^2^ | 26.4 | ^[10]^ |
| Aquivion, EW833 | 30% | 80 | 50 | 0.9 | 0.24 at 1A/cm^2^ | − | ^[11]^ |
| Aquivion EW720 | 0.65 | 80 | 30 | 1.15 | − | 28.7 | ^[12]^ |
| Aquivion, EW830 | 0.7 | 70 | 100 | 1.41 | 0.52 at 1A/cm^2^ | − | ^[13]^ |
| AQ830 | 32% | 80 | 100 | 1.03 | 0.6 at 0.6 V | 10.7, 80◦C  50% | ^[14]^ |
| AQ830 | 10% | 80 | 100 | 0.7 | 0.33 at 1A/cm^2^ | − | ^[15]^ |
| EW750 | 0.75 | 80 | 100 | 1.01 | − | 17 | ^[16]^ |

**Table S1** A comparative analysis of the performance with existing literature.

**Table S2** The summaries of O_2_ transport resistances

| Transport mechanism | Correlation | | Dominant factors |
| --- | --- | --- | --- |
|  | **Back pressure** | **humidity** |  |
| Molecular diffusion: R_MD_ | **√** | **×** | Large pores in GDL, CL |
| Knudsen diffusion: R_Kn_ | **×** | **×** | Small pores in GDL, CL |
| Permeation (dissolution and diffusion): R_ion_ | **×** | **√** | Ionomer in CL |

**Table S3** The methods to separate the O_2_ transport resistances.

| Step | Methods | Target |
| --- | --- | --- |
| 1 | To adjust the pressure | To separate the R_MD_ from total resistances |
| 2 | To adjust the humidity | To separate the R_ion_ from total resistances |

The relationship of the R_MD_, R_Kn_, and R_CL,ion_ is shown below^[4]^:

R_tot_= R_MD_ +**R_NP_**= R_MD_ + **R_Kn_ + R_CL,ion_** = aP +**b**

R_tot_= R_MD_ + R_Kn_ + **R_RH_** = R_MD_ + R_Kn_ + **R_CL,ion_** = d + **c*e^-kRH^**

**Table S4** The summaries of limiting current density (J_L_) of O_2_ transport experiment at various pressures and humidities for **Sample 1 # LSC-MEA**.

**J_L_ at various pressure:**

| **P abs. /kPa** | **J_1_** | **J_2_** | **J_3_** | **Average J_L_ / mA/cm^2^** |
| --- | --- | --- | --- | --- |
| 101 | 150.8 | 150.2 | 155.4 | 152.1 |
| 151 | 183.6 | 185.9 | 181.6 | 184.0 |
| 201 | 197.3 | 196.4 | 203.5 | 199.1 |
| 251 | 213.5 | 208.6 | 207.9 | 210.0 |
| 301 | 216.7 | 222.6 | 227.8 | 222.4 |

**Transport resistance at various pressure:**

| **P abs. /kPa** | **Average** **J_L_ / mA/cm^2^** | **P_H2O_ / Pa** | **C_O2_ / mol/m^3^** | **R_tot_/ s m^-1^** |
| --- | --- | --- | --- | --- |
| 101 | 152.1 | 22538.7 | 0.280 | 71.2 |
| 151 | 184.0 | 22538.7 | 0.458 | 96.1 |
| 201 | 199.1 | 22538.7 | 0.636 | 123.4 |
| 251 | 210.0 | 22538.7 | 0.814 | 149.6 |
| 301 | 222.4 | 22538.7 | 0.992 | 172.2 |

**J_L_ at various humidity:**

| **RH / %** | **J_1_** | **J_2_** | **J_3_** | **Average J_L_ / mA/cm^2^** |
| --- | --- | --- | --- | --- |
| 30 | 113.1 | 115.1 | 102.7 | 110.3 |
| 70 | 140.5 | 149.7 | 152.2 | 147.5 |
| 90 | 150.3 | 151.5 | 154.5 | 152.1 |

**Transport resistance at various humidity:**

| **RH / %** | **Average J_L_ / mA/cm^2^** | **P_H2O_ / Pa** | **C_O2_ / mol/m^3^** | **R_tot_ / s m^-1^** |
| --- | --- | --- | --- | --- |
| 30 | 110.3 | 7512.9 | 0.334 | 117.1 |
| 70 | 147.5 | 17530.1 | 0.298 | 78.4 |
| 90 | 152.1 | 22538.7 | 0.280 | 71.2 |

**Table S5** The summaries of limiting current density of O_2_ transport experiment at various pressures and humidities for **Sample 2 # SSC-MEA**.

**J_L_ at various pressure:**

| **P abs. /kPa** | **J_1_** | **J_2_** | **J_3_** | **Average**  **J_L_ / mA/cm^2^** |
| --- | --- | --- | --- | --- |
| 101 | 211.5 | 196.1 | 204.4 | 204.0 |
| 151 | 250.8 | 235.2 | 267.0 | 251.0 |
| 201 | 260.4 | 253.2 | 246.4 | 253.3 |
| 251 | 272.7 | 269.8 | 254.5 | 265.7 |
| 301 | 269.9 | 288.1 | 263.0 | 273.7 |

**Transport resistance at various pressure:**

| **P abs. /kPa** | **Average J_L_ / mA/cm^2^** | **P_H2O_ / Pa** | **C_O2_ / mol/m^3^** | **R_tot_/ s m^-1^** |
| --- | --- | --- | --- | --- |
| 101 | 204.0 | 22538.7 | 0.280 | 53.0 |
| 151 | 251.0 | 22538.7 | 0.458 | 70.5 |
| 201 | 253.3 | 22538.7 | 0.636 | 96.9 |
| 251 | 265.7 | 22538.7 | 0.814 | 118.2 |
| 301 | 273.7 | 22538.7 | 0.992 | 139.9 |

**J_L_ at various humidity:**

| **RH / %** | **J_1_** | **J_2_** | **J_3_** | **Average J_L_ / mA/cm^2^** |
| --- | --- | --- | --- | --- |
| 30 | 94.6 | 94.5 | 94.6 | 94.6 |
| 70 | 179.1 | 184.5 | 184.8 | 182.8 |
| 90 | 211.0 | 198.0 | 203.0 | 204.0 |

**Transport resistance at various humidity:**

| **RH / %** | **Average J_L_ / mA/cm^2^** | **P_H2O_ / Pa** | **C_O2_ / mol/m^3^** | **R_tot_/ s m^-1^** |
| --- | --- | --- | --- | --- |
| 30 | 94.6 | 7512.9 | 0.334 | 136.2 |
| 70 | 182.8 | 17530.1 | 0.298 | 63.0 |
| 90 | 204.0 | 22538.7 | 0.280 | 53.1 |

Reference

[1] P. Guan, Y. Zou, M. Zhang, W. Zhong, J. Xu, J. Lei, H. Ding, W. Feng, F. Liu, Y. Zhang, *Science Advances* **2023**, 9, eadh1386.

[2] N. Koumakis, G. Petekidis, *Soft Matter* **2011**, 7, 2456.

[3] M. Li, F. Liu, S. Pei, Z. Zhou, K. Niu, J. Wu, Y. Zhang, *Nanomaterials* **2023**, 13, 444.

[4] N. Nonoyama, S. Okazaki, A. Z. Weber, Y. Ikogi, T. Yoshida, *Journal of The Electrochemical Society* **2011**, 158, B416.

[5] R. Makharia, M. F. Mathias, D. R. Baker, *Journal of The Electrochemical Society* **2005**, 152, A970.

[6] M. Cimenti, D. Bessarabov, M. Tam, J. Stumper, *ECS transactions* **2010**, 28, 147.

[7] H. Sun, *The Journal of Physical Chemistry B* **1998**, 102, 7338.

[8] Q. Zhang, S. Dong, P. Shao, Y. Zhu, Z. Mu, D. Sheng, T. Zhang, X. Jiang, R. Shao, Z. Ren, *Science* **2022**, 378, 181.

[9] H. Ren, Y. Teng, X. Meng, D. Fang, H. Huang, J. Geng, Z. Shao, *Journal of Power Sources* **2021**, 506, 230186.

[10] S. Liu, R. Lin, J. Lu, Y. Wang, X. Cai, *Chemical Engineering Journal* **2023**, 472, 145050.

[11] K. Talukdar, P. Gazdzicki, K. A. Friedrich, *Journal of Power Sources* **2019**, 439, 227078.

[12] H. Ren, X. Meng, Y. Lin, Z. Shao, *Journal of Power Sources* **2022**, 517, 230698.

[13] W. Gao, Q. Yin, J. Chen, Z. Liu, Z. Zhang, J. Lu, Y. Lei, H. Xu, H. Ouyang, Y. Yin, *Chemical Engineering Journal* **2024**, 479, 147787.

[14] Y. Garsany, R. W. Atkinson, M. B. Sassin, R. M. Hjelm, B. D. Gould, K. E. Swider-Lyons, *Journal of The Electrochemical Society* **2018**, 165, F381.

[15] C. Lei, D. Bessarabov, S. Ye, Z. Xie, S. Holdcroft, T. Navessin, *Journal of Power Sources* **2011**, 196, 6168.

[16] S. Shen, A. Han, X. Yan, J. Chen, X. Cheng, J. Zhang, *Journal of The Electrochemical Society* **2019**, 166, F724.
